# Numerically analysis of Marangoni convective flow of hybrid nanofluid over an infinite disk with thermophoresis particle deposition

**DOI:** 10.1038/s41598-023-32011-x

**Published:** 2023-03-28

**Authors:** Munawar Abbas, Nargis Khan, M. S. Hashmi, Jihad Younis

**Affiliations:** 1 Department of Mathematics, The Islamia University of Bahawalpur, Bahawalpur, Pakistan; 2 Department of Mathematics, The Govt. Sadiq College Women University, Bahawalpur, Pakistan; 3 Department of Mathematics, Aden University, P.O. Box 6014, Aden, Yemen

**Keywords:** Applied mathematics, Computational science

## Abstract

This study discusses the flow of hybrid nanofluid over an infinite disk in a Darcy–Forchheimer permeable medium with variable thermal conductivity and viscosity. The objective of the current theoretical investigation is to identify the thermal energy characteristics of the nanomaterial flow resulting from thermo-solutal Marangoni convection on a disc surface. By including the impacts of activation energy, heat source, thermophoretic particle deposition and microorganisms the proposed mathematical model becomes more novel. The Cattaneo-Christov mass and heat flux law is taken into account when examining the features of mass and heat transmission rather than the traditional Fourier and Fick heat and mass flux law. MoS_2_ and Ag nanoparticles are dispersed in the base fluid water to synthesize the hybrid nanofluid. PDEs are transformed to ODEs by using similarity transformations. The RKF-45th order shooting method is used to solve the equations. With the use of appropriate graphs, the effects of a number of non-dimensional parameters on velocity, concentration, microorganism, and temperature fields are addressed. The local Nusselt number, density of motile microorganisms and Sherwood number are calculated numerically and graphically to derive correlations in terms of the relevant key parameters. The findings show that as we increase the Marangoni convection parameter, skin friction, local density of motile microorganisms, Sherwood number, velocity, temperature and microorganisms profiles increase, whereas Nusselt number and concentration profile exhibit an opposite behavior. The fluid velocity is reduced as a result of enhancing the Forchheimer parameter and Darcy parameter.

## Introduction

Marangoni convection is commonly defined as the edge dissipative layer between two phase fluid flows, such as liquid–liquid and gas–liquid interfaces. It is subjected to surface tension variations caused by changes in chemical concentration, temperature and applied magnetic fields. The presence of different fluids at interface which have distinct fluid characteristics may causes these gradients appear. External forces like gravitational and shear forces are activated as a result of the viscosity of interacting liquids. Due to their extensive usage in the disciplines of space processing, microgravity science and industrial manufacturing procedures, governing equations have drawn the attention of the majority of researchers who are interested in modeling these external forces. The importance of thermo-solutal Marangoni convection flows in the procedure of mass and heat transfer into various schemes has been carefully examined in^1–5^$.$ Chemical reaction in the thermo-solutal Marangoni convective flow across the Riga plate was studied by Shafiq et al.^6^. The encouragements of heat radiation on Marangoni convective flow were examined by Hayat et al.^7^$.$ Microorganisms that are typically 5–10% denser than water swim upward in fluid flow known as bio-convection. By increasing the base fluid density in a specific direction, the self-impelled microorganisms cause bio-convection in the flow. The motile microbes can be characterized into many types of microorganisms, such as oxytocic, gyrotactic, and negative gravitaxis microorganisms, on the basis of impellent. Negative gravity, a gradient in oxygen content, and a distinction between buoyant heat and mass all act as stimuli for these microbes. Motile microorganisms improve the concentration or mass transfer rate of species in the suspension, which has industrial uses in enzyme biosensors, polymer sheets, chemical processing, and biotechnological applications. Waqas et al.^8^ inspected the effect of bio-convection on second-grade nanofluid microorganisms. For further details see^9–12^$.$

**Figure 1:**
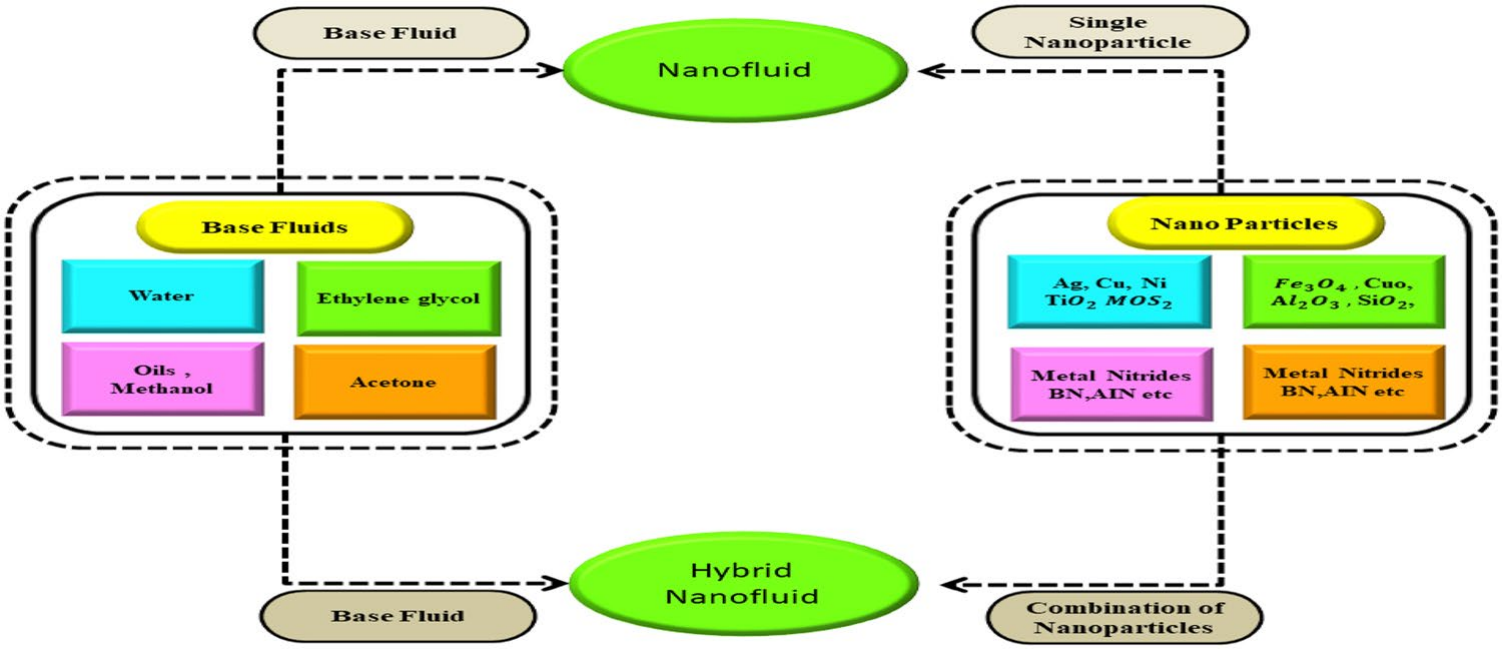
Hybrid nanofluid and nanofluid manufacturing process.

Nanoparticles with a diameter of 1–100 nm suspended in a base fluid make up nanofluids. A fluid containing a nanoscale particle is referred to as nanoliquid. The physical features of base liquids, such as their density, thermal conductivity, viscosity, and electrical conductivity, continue to be affected by nanoparticles. Nanofluids are an important component of nanomaterials and are used in a variation of industrial processes, including smart computers, solar panels, renewable energy materials, optics, electronics, and catalysis. According to Madhukesh et al.^13^, slip influences and the Cattaneo-Christov theory have an impact on the hydromagnetic micropolar-casson nanofluid flow over porous disc. Regarding the MHD thermal radiative heat transfer of nanofluid over a flat plate in a porous media in the presence of chemical reaction and variable thermal conductivity, consider Pal and Mandal^14^$.$ A lot of researchers^15–22^ have focused on nanofluid flow and its industrial and nuclear applications. A unique type of nanofluid known as a hybrid nanoliquid occurs when two different nanoscale particles are dispersed in the fluid in a variety of ways. In order to incorporate the positive effects of both nanomaterials into a single stable homogenous system, various nanomaterial configurations were considered. A hybrid nanofluid is a rapidly developing field. Hybrid electrical systems, modern automated cooling systems, fuel cells, car heat dissipation, bio-medicine manufacturing, gas sensing, renewable electricity, domestic freezers and transistors are just a few of the applications of hybrid nanoliquids. The impacts of connective flow of Maxwell hybrid nanofluid passing through the channel were investigated by Ali et al.^23^$.$ The context^24–27^ highlights a few of the important and interesting studies. Figure 1 shows hybrid nanofluid and nanofluid manufacturing process.

The thermophoresis phenomenon is beneficial in numerous micro-engineering and industrial applications, especially for protecting cleaning gas, preventing micro-contamination, nuclear reactors and preventing heat exchanger corrosion. This marvel happens when a combination of several moveable particles is exposed to a temperature variance the various particle kinds react in various ways. Thermophoresis permits small particles to deposit on cold surfaces and allows them to migrate away from heated surfaces. A thermophoretic force is the force caused by the temperature difference that the suspended particles perceive. Gradually, the concept of thermophoresis developed from Goren^28^ examination of the theoretical problem of aerosol particle thermophoresis in the laminar compressible boundary layer flow on a flat plate. Alam et al.^29^ examine the thermophoresis particle deposition on temporary forced convective flow caused by a rotating disc. Authors^30–36^ analyzed the flow with various geometries by taking thermophoretic deposition of particles into account. The heat generation/absorption outcome on the heat transmission is another amazing aspect to take into account in a variety of real-world problems. In the MHD flow with a heat generation, Shi et al.^37^ analyzed mass and heat transmission in the radiative Maxwell nanofluid. The heat generation properties on the Maxwell nano liquid flow through an expanded cylinder were deliberated by Irfan et al.^38^. Basha et al.^39^ inspected how the flow of chemically reacting nanoliquid gets affected by the heat source.

**Figure 2:**
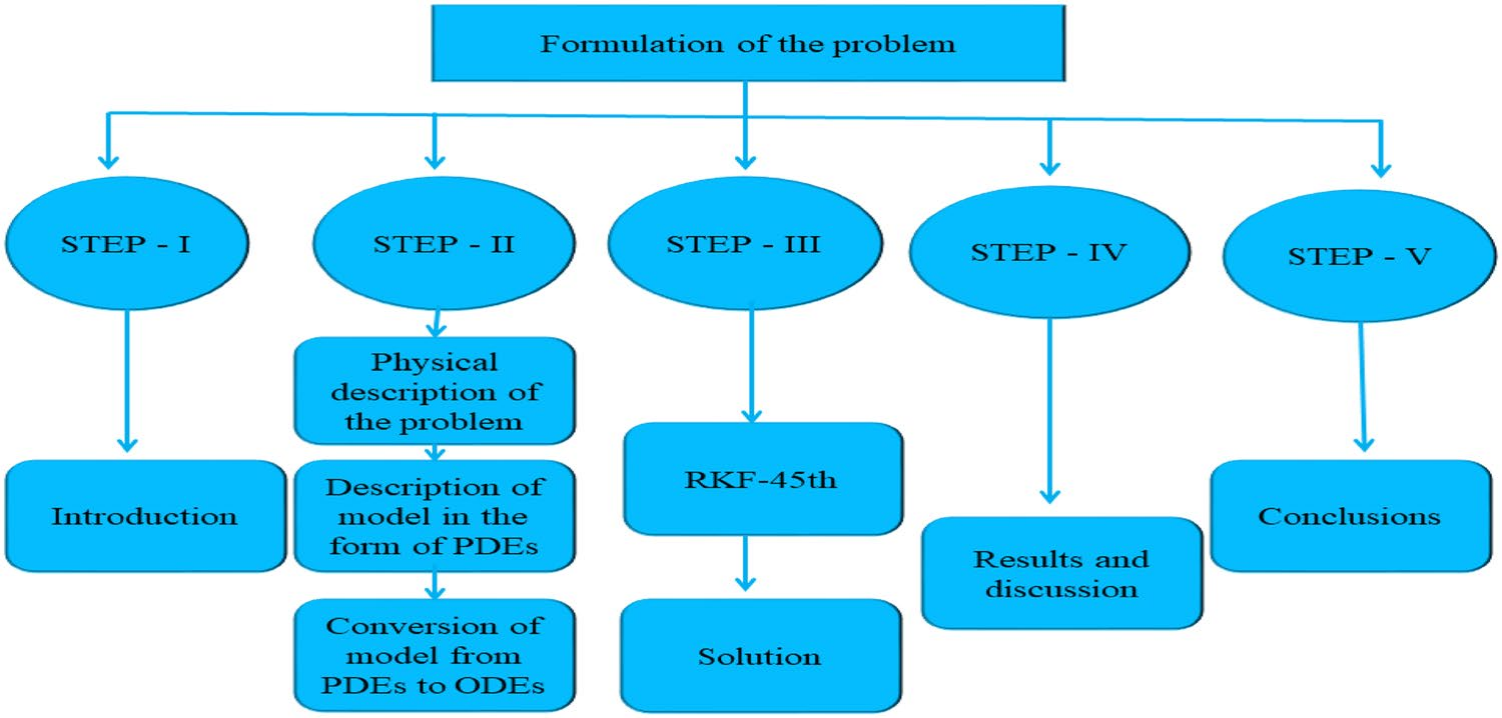
Formulation of the problem.

When two different types of bodies have different temperatures, the heat transfer mechanism takes place. Mass and heat transfer mechanisms are used in a variation of processes, including the casting of metals, latent heat storage, the production of polyethylene and paper, crystal growth, nuclear reactor cooling and biomedical applications like tissue drug targeting. Fourier^40^ and Fick^41^ were the first to explain the processes of mass and heat transmission. They assert that parabolic equations exist for temperature and concentration distributions. Later, Cattaneo^42^ modified Fourier's law of heat conduction by incorporating the thermal relaxation factor; as a result, he examined heat transmission with a limited speed in thermal waves. In order to achieve the material-invariant formulation, Christov^43^ proposed a novel model that substitutes Oldroyd's upper-convected derivative for the time derivative. Figure 2 displays flow chart of the problem.

**Figure 3:**
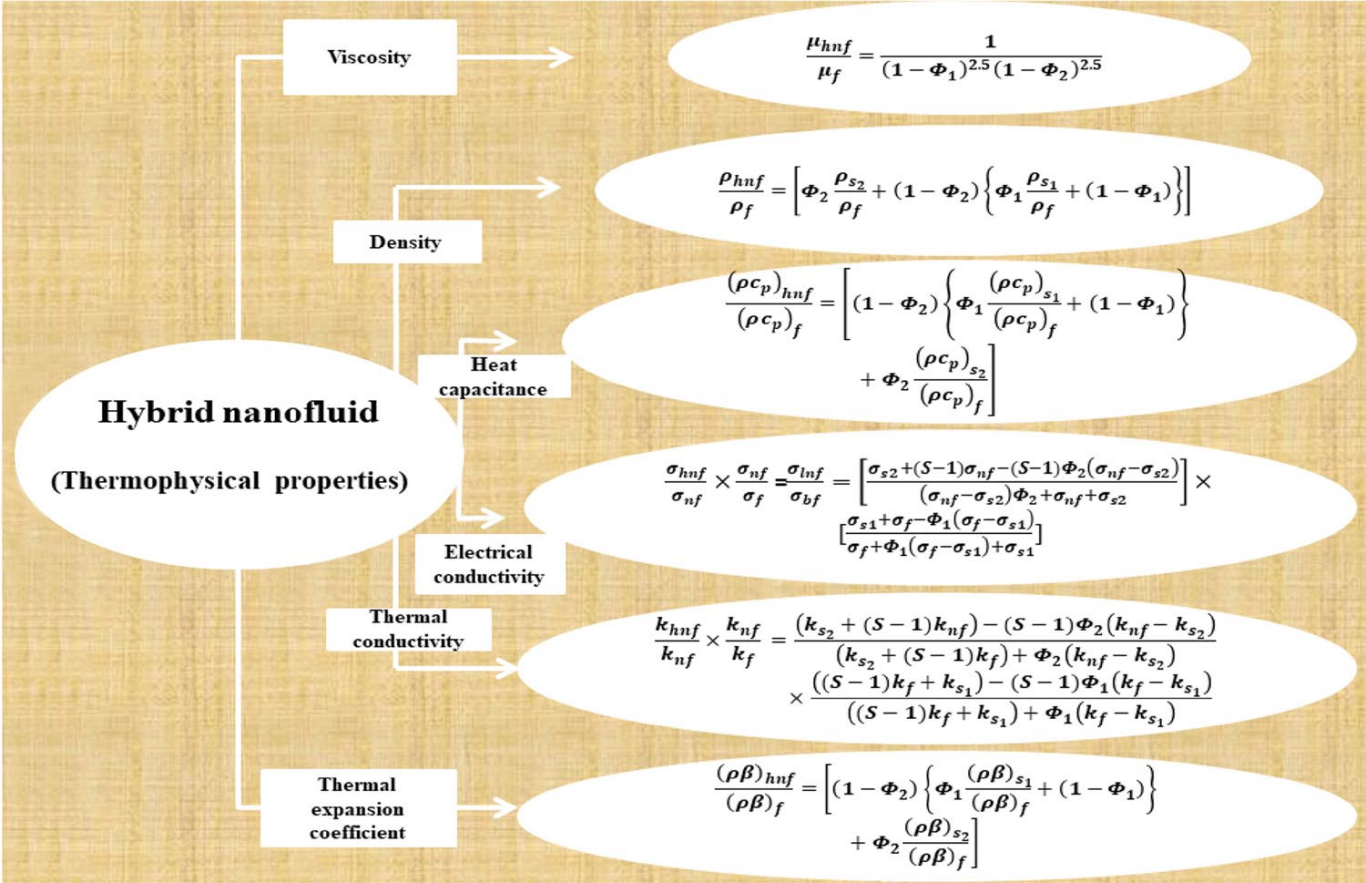
Thermo-physical properties of the hybrid-nanofluid.

The primary goal of our investigation is to explore the heat energy characteristics of Darcy–Forchheimer flow of hybrid nanofluid with thermo-solutal Marangoni convection over an infinite disc with activation energy and Joule heating. The flow problem concentration and energy equations are regulated by the Cattaneo-Christov model. The study of the modified Fourier and Fick heat and mass flux model in the thermo-solutal Marangoni convective flow of hybrid nanoparticles is the main novel idea. Visual representations of numerical solutions are discussed via graphs. The rates of local heat, mass and microorganism transfer are discussed and analyzed in the form of tables. Figure 3 shows thermo-physical properties of the hybrid-nanofluid. The current investigation employs numerical and statistical techniques to address the following questions.

- What impacts are observed on the temperature, microorganisms, velocity and concentration profiles in the presence of Marangoni convection parameter?
- How does the hybrid nanofluid influence the rates of mass and heat transfer?
- What effects does thermal and solutal relaxation parameter have on the profiles of concentration and temperature?
- What are the influences of nanoparticles volume fraction parameters on thermal and velocity profiles?
- How will the non-uniform heat source affect the temperature profile?
- How does the concentration profile changes as a result of the thermophoretic and activation energy parameters?

## Mathematical formulation

**Figure 4:**
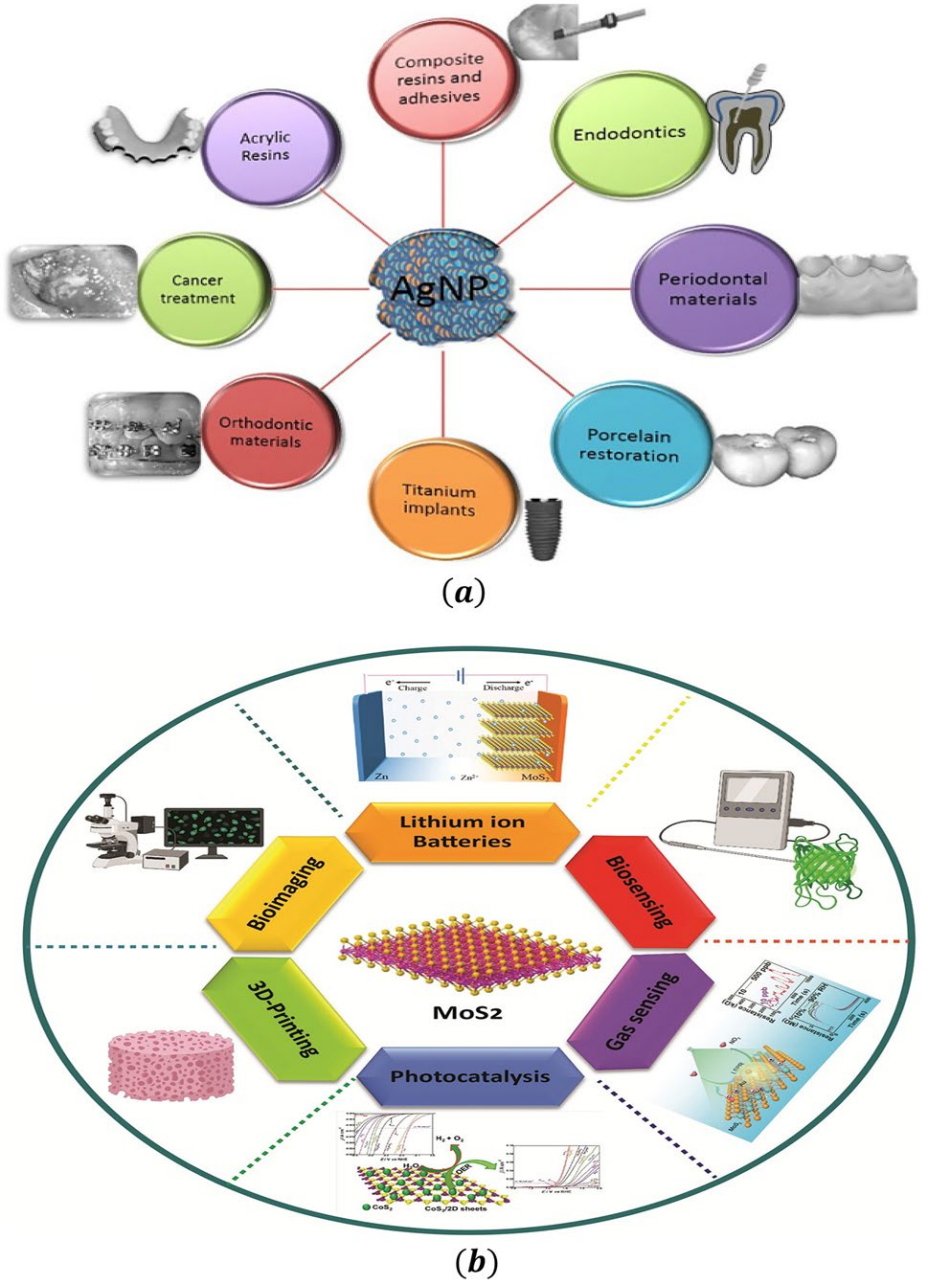
(**a**) Applications of silver nanoparticle. (**b**) Applications of molybdenum disulphide (MoS_2_) nanoparticle.

**Figure 5:**
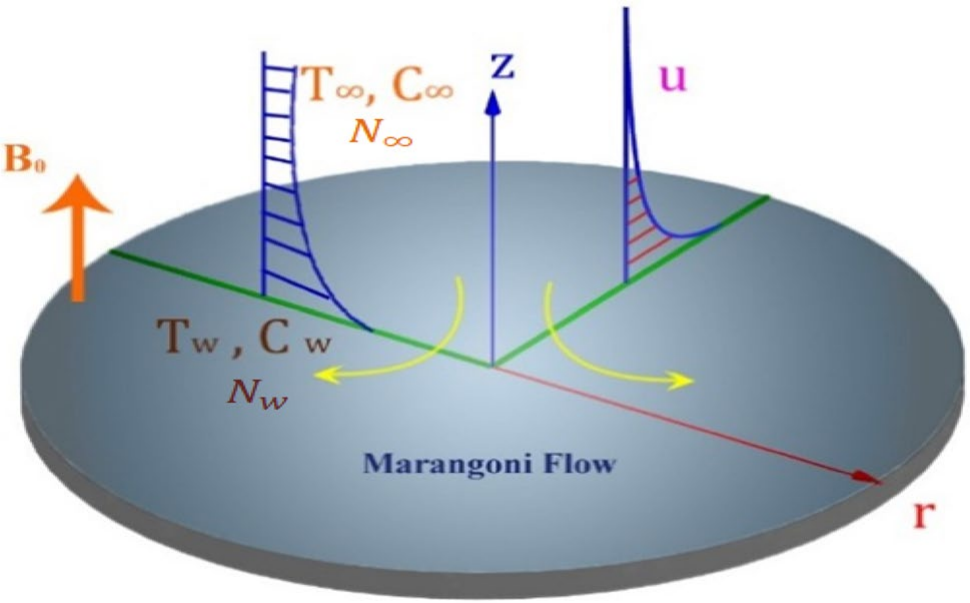
Physical model.

The applications of silver and molybdenum disulfide nanoparticles is given in Fig. 4 (a,b). We have considered surface-tension-driven hybrid nanofluid flow over an infinite disc. Figure 5 depicts the physical model of the problem in cylindrical coordinate $(r, \phi , z)$ system. The flow is motivated by the Marangoni layer a surface tension caused by the surface temperature, and is symmetric to $z = 0$ plane and axisymmetric around the z-axis with $\frac{\partial }{\partial \phi }= 0$ for all variables. The fluid is considered to be incompressible and electrically conducting, and the flow is supposed to be steady, laminar and irrational. The magnetic field is applied along $z$-direction. Hybrid nanofluid synthesizes using the silver (Ag) and molybdenum disulphide (MoS_2_) particles with water (H_2_O) as base fluid.

### Assumptions of the model

The following presumptions and conditions are applied when analyzing the mathematical model.

- Darcy–Forchheimer flow
- Activation energy is analyzed
- Non-uniform heat source is assumed
- Variable thermal diffusivity is investigated
- Viscous dissipation and Joule heating is addressed
- The Cattaneo-Christov mass and heat flux is discussed
- The size of nanoparticles is uniform whereas the shape is spherical
- Gyrotactic microorganisms and themophoretic particles are used (Fig. 5).

### Basic governing equations

The governing flow equations are (Mackolil et al.^44^, Mahanthesh et al.^45^ and Basavarajappa et al.^46^):

1 $$\frac{\partial u}{\partial r}+\frac{u}{r}+\frac{\partial w}{\partial z}=0,$$

2 $${\rho }_{hnf}\left(u\frac{\partial u}{\partial r}+w\frac{\partial u}{\partial z} \right)=\frac{\partial }{\partial z}\left({\mu }^{*}\left(\text{T}\right)\frac{\partial u}{\partial \text{z}}\right)-{\sigma }_{hnf}{B}_{0}^{2}u-{\mu }^{*}\left(\text{T}\right)\frac{u}{{k}_{1}}-\frac{{C}_{b}}{\sqrt{{k}_{1}}}{u}^{2},$$

3 $$\begin{aligned} \left( {\rho c_{p} } \right)_{{hnf}} \left( {u\frac{{\partial T}}{{\partial r}} + w\frac{{\partial T}}{{\partial z}}} \right) & = \frac{\partial }{{\partial z}}\left( {K^{*} \left( {\text{T}} \right)\frac{{\partial T}}{{\partial z}}} \right) - \frac{{\partial q_{r} }}{{\partial z}} + \frac{{K^{*} \left( {\text{T}} \right)\rho _{{hnf}} U_{0} }}{{\mu _{{hnf}} }}[A^{*} (T_{w} - T_{\infty } )f^{\prime} + B^{*} \left( {T - T_{\infty } } \right)] \\ & \quad - \lambda _{T}^{*} + \mu ^{*} \left( {\text{T}} \right)\left( {\frac{{\partial u}}{{\partial z}}} \right)^{2} + \sigma _{{hnf}} B_{0}^{2} u^{2} , \\ \end{aligned}$$

4 $$u\frac{\partial C}{\partial r}+w\frac{\partial C}{\partial z}=\frac{\partial }{\partial \text{z}}\left({{D}_{B}}^{*}(C)\frac{\partial C}{\partial \text{z}}\right)-\frac{\partial }{\partial \text{z}}(\frac{{k}^{*}{v}_{f}}{{T}_{ref}}\frac{\partial T}{\partial y} \left(C-{C}_{\infty }\right)-{k}_{r}^{2}(C-{C}_{\infty })(\frac{T}{{T}_{\infty }}{)}^{m}\text{exp}\left(\frac{-Ea}{KT}\right)-{\lambda }_{C}^{*},$$

5 $$u\frac{\partial N}{\partial r}+w\frac{\partial N}{\partial z}={D}_{m}\frac{{\partial }^{2}N}{\partial {z}^{2}}-\frac{b{W}_{c}}{{C}_{w}-{C}_{\infty }}\frac{\partial }{\partial \text{z}}\left( N\frac{\partial C}{\partial z} \right).$$

The concentration and thermal diffusion with mass and heat flow relaxation are described by the Cattaneo-Christov double diffusion theory.

6 $${{\lambda }_{C}^{*}=\lambda }_{c}\left[{u}^{2}\frac{{\partial }^{2}C}{\partial {r}^{2}}+{w}^{2}\frac{{\partial }^{2}C}{\partial {z}^{2}}+2uw\frac{{\partial }^{2}C}{\partial r\partial z}+\frac{\partial C}{\partial r}\left(u\frac{\partial u}{\partial r}+w\frac{\partial u}{\partial z}\right)+\frac{\partial C}{\partial z}\left(u\frac{\partial w}{\partial r}+w\frac{\partial w}{\partial z}\right)\right],$$

7 $${{\lambda }_{T}^{*}=\lambda }_{T}\left[{u}^{2}\frac{{\partial }^{2}T}{\partial {r}^{2}}+{w}^{2}\frac{{\partial }^{2}T}{\partial {z}^{2}}+2uw\frac{{\partial }^{2}T}{\partial r\partial z}+\frac{\partial T}{\partial r}\left(u\frac{\partial u}{\partial r}+w\frac{\partial u}{\partial z}\right)+\frac{\partial T}{\partial z}\left(u\frac{\partial w}{\partial r}+w\frac{\partial w}{\partial z}\right)\right].$$

The appropriate boundary conditions^46^:

8 $$\mu ^{*} \left( {\text{T}} \right)\frac{{\partial u}}{{\partial z}} = \frac{{\partial \sigma _{1} }}{{\partial r}} = - \left( {\frac{{\partial \sigma _{1} }}{{\partial T}}\frac{{\partial T}}{{\partial r}} + \frac{{\partial \sigma _{1} }}{{\partial C}}\frac{{\partial C}}{{\partial r}}} \right),\quad w = 0,\;{\text{at}}\;z = 0,$$

9 $$T = T_{w} = T_{\infty } + B_{1} {\text{r}}^{2} ,C = C_{w} = C_{\infty } + B_{2} {\text{r}}^{2} ,\quad {\text{at}}\;z = 0,$$

10 $$N = N_{w} = N_{\infty } + B_{2} {\text{r}}^{2} \quad {\text{at}}\;z = 0,$$

11 $$u \to 0,\;T \to T_{\infty } ,\;C \to C_{\infty },\;N \to N_{\infty } \quad {\text{at}}\;z \to \infty ,$$

12 $${\sigma }_{1}={\sigma }_{0}\left(1-{\gamma }_{T}\left(T-{T}_{\infty }\right)-{\gamma }_{C}\left(C-{C}_{\infty }\right)\right), {\gamma }_{T}=-{\left.\frac{1}{{\sigma }_{0}}\frac{\partial {\sigma }_{1}}{\partial T}\right|}_{T},{\gamma }_{C}=-{\left.\frac{1}{{\sigma }_{0}}\frac{\partial {\sigma }_{1}}{\partial C}\right|}_{C}.$$

### Non-uniform heat source

The term ${\text{q}}^{{\prime \prime \prime }}$ known as the non-uniform heat generation/absorption can be defined as (Obalalu et al.^47^)

13 $$q^{{\prime \prime \prime }} = \frac{{K^{*} ({\text{T}})\rho _{{hnf}} U_{0} }}{{2\mu ^{*} ({\text{T}}))X}}\left[ {A^{*} (T_{w} - T_{\infty } )f^{\prime} + B^{*} \left( {T - T_{\infty } } \right)} \right].$$

### Variable viscosity

The suggested temperature dependent viscosity is given as (Ghaly et al.^48^).

14 $${{\mu }^{*}\left(\text{T}\right)=\mu }_{\text{hnf }}{e}^{-{\varepsilon }_{1}\left(\frac{\text{T}-{\text{T}}_{\infty }}{{\text{T}}_{\text{w}}-{\text{T}}_{\infty }}\right)}.$$

### Variable thermal conductivity

The thermal conductivity that is temperature-dependent can be defined as (Obalalu et al.^47^).

15 $${K}^{*}\left(\text{T}\right)={k}_{\text{hnf}}\left[1+{\varepsilon }_{2}\left(\frac{T-{T}_{\infty }}{{T}_{w}-{T}_{\infty }}\right)\right].$$

### Variable concentration relation

It is presumed that the diffusivity coefficient follows a linear function. This corresponds to the description of (Obalalu et al.^47^).

16 $${{D}_{B}}^{*}\left(C\right)={\text{D}}_{\text{B}}\left[1+{\varepsilon }_{3}\left(\frac{\text{C}-{\text{C}}_{\infty }}{{\text{C}}_{\text{w}}-{\text{C}}_{\infty }}\right)\right].$$

### Similarity transformation

Now take into consideration the aforementioned Von Karman transformations (Karman et al.^49^):

17 $$u=r{\Omega }^{*}F\left(\xi \right), \quad w=\sqrt{{\Omega }^{*}v}H\left(\xi \right), \quad T={T}_{\infty }+{B}_{1}{\text{r}}^{2}\theta \left(\xi \right),$$

18 $$C={C}_{\infty }+{B}_{2}{\text{r}}^{2}\phi \left(\xi \right), \quad N={N}_{\infty }+{\text{r}}^{2}{B}_{3}\Theta \left(\xi \right), \quad \xi =\text{z}\sqrt{\frac{{\Omega }^{*}}{\nu }}.$$

### Non-dimensional model

The driving PDEs are transformed into ODEs using the similarity variables.

19 $$2F + H^{\prime} = 0,$$

20 $$A_{1} ( - \varepsilon _{1} F^{\prime}\theta ^{\prime} + {\text{e}}^{{ - \theta \varepsilon _{1} }} F^{{\prime \prime }} ) - A_{3} FM - A_{1} Ke^{{ - \theta \varepsilon _{1} }} F - F^{2} F_{r} - A_{2} (F^{2} + F^{\prime}H) = 0,$$

21 $$\begin{aligned} & \left( {A_{5} \left( {\theta ^{{\prime 2}} \varepsilon _{2} + \left( {1 + \varepsilon _{2} \theta } \right)\theta ^{\prime\prime}} \right) + {\text{Rd}}\theta ^{\prime\prime} + \frac{{A_{5} \left( {1 + \varepsilon _{2} \theta } \right)A_{2} }}{{A_{1} e^{{ - \theta \varepsilon _{1} }} }}\left( {A^{*} F + B^{*} \theta } \right) + \Pr {\text{Ec}}A_{1} e^{{ - \theta \varepsilon _{1} }} (F^{\prime})^{2} } \right. \\ & - A_{3} \Pr {\text{EcM}}({\text{F}})^{2} - \gamma _{1} \Pr (4F^{2} {{\uptheta }} + H^{2} \theta ^{\prime\prime} + 4{\text{HF}}\theta ^{\prime} + 2{\text{HF}}^{\prime } {{\uptheta }} + \theta ^{\prime}{\text{HH}}^{\prime } ) \\ & - A_{6} \Pr (2F\theta + H{{\uptheta }}^{\prime } ) = 0, \\ \end{aligned}$$

22 $$\begin{aligned} & \left( {\varepsilon _{3} \phi ^{{\prime 2}} + \left( {1 + \varepsilon _{3} \phi } \right)\phi ^{\prime\prime}} \right) - {\text{Sc}}\left( {2F\phi + H\phi ^{\prime}} \right) - \gamma _{2} {\text{Sc}}\left( {4F^{2} \upphi + H^{2} \phi ^{\prime\prime} + 4{\text{HF}}\phi ^{\prime} + 2{{{\text {HF}}^{\prime}}}\upphi } \right. \\ & \quad \left. { + \phi ^{\prime}{\text{HH}}^{\prime}} \right) - ScRc(1 + \delta \theta )^{m} \exp \left( {\frac{{ - E}}{{1 + \delta \uptheta }}} \right)\upphi - \uptau {\text{Sc}}({{\uptheta ^{\prime}}}\upphi ^{\prime} + \upphi {{\uptheta ^{\prime\prime}}}) = 0, \\ \end{aligned}$$

23 $$\Theta ^{\prime\prime} - {\text{Sb}}(2F\Theta + H\Theta ^{\prime}) - Pe(\Theta ^{\prime}\phi ^{\prime} + \left( {\sigma + \Theta } \right)\phi ^{\prime\prime} = 0.$$

Boundary conditions

24 $$A_{1} e^{{ - \theta \varepsilon _{1} }} F^{\prime}\left( 0 \right) = - 2Mn\left( {1 + Ma} \right),H\left( 0 \right) = 0,\theta \left( 0 \right) = 1,\phi \left( 0 \right) = 1,\Theta \left( 0 \right) = 1,$$

25 $$F\left(\infty \right)\to 0, \theta \left(\infty \right)\to 0, \phi \left(\infty \right)\to 0,\Theta (\infty )\to 0.$$

### Expressions of parameters

The following are the non-dimensional parameters: $Pr=\frac{{\upsilon }_{f}\left(\rho {c}_{p}\right)}{{k}_{f}}$ is the Prandtl number, $\delta = \frac{{{r}^{2}B}_{2}}{{T}_{\infty }}$ is the temperature difference parameter, $\tau =\frac{{k}^{*}{v}_{f}{r}^{2}{B}_{1}}{{\upsilon }_{f}{\rho }_{f}{T}_{f}}$ is the Thermophoretic parameter, $M=\frac{{\sigma }_{f}{B}_{0}^{2}}{{\Omega }^{*}{\rho }_{f}}$ is the magnetic parameter, ${F}_{r}=\frac{{rc}_{b}}{\sqrt{{k}_{1}}}$ is the Forchheimer parameter, $Rc=\frac{{k}_{r}^{2}}{{\Omega }^{*}}$ is the chemical reaction parameter, $E=\frac{{E}_{a}}{{{T}_{\infty }K}^{*}}$ is the activation energy parameter, $K=\frac{{\upsilon }_{f}}{{{\Omega }^{*}k}_{1}}$ is the Inverse Darcy parameter, $\text{Ec}=\frac{{v}_{f}{{\Omega }^{*}}^{2}}{{c}_{p}{B}_{1}}$ is the Eckert number, $Rd=\frac{{16\sigma }^{*}{T}_{\infty }^{3}}{3{{k}^{+}k}_{f}}$ is the thermal radiation parameter, $Sc =\frac{{\upsilon }_{f}}{{D}_{B}}$ is the Schmidt number, $Ma=\frac{{\gamma }_{C}{B}_{2}}{{\gamma }_{T}{B}_{1}}$ is the Marangoni ratio number, $Mn=\frac{{\sigma }_{0}{\gamma }_{T}{rB}_{1}}{{\mu }_{f} {\Omega }^{*}}\sqrt{\frac{v}{{\Omega }^{*}}}$ is the Marangoni number, ${\gamma }_{1}={{\Omega }^{*}\lambda }_{T}$ is the thermal relaxation parameter, and ${\gamma }_{2}={{\Omega }^{*}\lambda }_{c}$ is the solutal relaxation parameter, $Pe=\frac{b{W}_{c}}{{D}_{m}}$ is the Peckelt number, $\sigma =\frac{{N}_{\infty }}{{{r}^{2}B}_{3}}$ is the Microorganisms difference parameter and $Sb=\frac{{\upsilon }_{f}}{{D}_{m}}$ is the bio-convection Schmidt number.

## Physical quantities

The following are the local Sherwood number $(S\text{h})$, Nusselt number $(N\text{u})$, density of motile microorganisms $(N\text{n})$ and skin friction $(C{f}_{x})$ (Basavarajappa et al. ^46^).

26 $$S{h}_{r}=\frac{-r{{D}_{B}}^{*}(C){\left(\frac{\partial C}{\partial z}\right)}_{z=0}}{{C}_{w}-{C}_{\infty }},$$

27 $$N{\text{u}}_{r}=\frac{-r({K}^{*}\left(\text{T}\right){+\frac{16{\sigma }^{*}{\text{T}}_{\infty }^{3}}{3{k}^{+}})\left(\frac{\partial T}{\partial z}\right)}_{z=0}}{{T}_{w}-{T}_{\infty }},$$

28 $$N{\text{n}}_{r}=\frac{-r{\left(\frac{\partial N}{\partial z}\right)}_{z=0}}{{N}_{w}-{N}_{\infty }}, \quad Cf=\frac{{\mu }^{*}\left(\text{T}\right){\left(\frac{\partial u}{\partial \text{z}}\right)}_{z=0}}{{\Omega }^{*}},$$

29 $$N{\text{u}} = \frac{{Nu_{r} }}{{\sqrt {Re} }} = - (A_{5} (1 + \varepsilon _{2} \theta (0)) + Rd\theta ^{\prime}\left( 0 \right),$$

30 $$S{\text{h}} = \frac{{Sh_{r} }}{{\sqrt {Re} }} = - \left( {1 + \varepsilon _{3} \phi \left( 0 \right)} \right)\phi ^{\prime}\left( 0 \right),$$

31 $$N{\text{n}} = \frac{{N{\text{n}}_{r} }}{{\sqrt {Re} }} = - \Theta \left( 0 \right),Cf_{x} = \frac{{Cf}}{{\sqrt {Re} }} = A_{1} e^{{ - \theta (0)\varepsilon _{1} }} F^{\prime}(0)$$

where $Re=\frac{{r}^{2}{\Omega }^{*}}{v}$ is the local Reynolds number. Table 1 displays thermo-physical characteristics of hybrid nanofluids. Table 2 shows the thermo-physical properties of regular fluids and nanoparticles.

**Table 1:** Hybrid nanofluid thermo-physical features (Nazir et al.^50^ and Khan et al.^51^).

Properties	Hybrid nanofluid
$\left({\mu }_{hnf}\right)$—dynamic viscosity	${A}_{1}=\frac{{\mu }_{hnf}}{{\mu }_{f}}=\frac{1}{{\left(1-{\Phi }_{1}\right)}^{2.5}{\left(1-{\Phi }_{2}\right)}^{2.5}}$
$\left({\rho }_{hnf}\right)$—density	${A}_{2}=\frac{{\rho }_{hnf}}{{\rho }_{f}}=\left[{\Phi }_{2}\frac{{\rho }_{{s}_{2}}}{{\rho }_{f}}+\left(1-{\Phi }_{2}\right)\left\{{\Phi }_{1}\frac{{\rho }_{{s}_{1}}}{{\rho }_{f}}+\left(1-{\Phi }_{1}\right)\right\}\right]$
$\left({\sigma }_{hnf}\right)$—electrical conductivity	${A}_{3}=\frac{{\sigma }_{hnf}}{{\sigma }_{nf}}\times \frac{{\sigma }_{nf}}{{\sigma }_{f}}$=$\frac{{\sigma }_{\text{lnf}}}{{\sigma }_{bf}}=\left[\frac{{\sigma }_{s2}+(\text{S}-1){\sigma }_{nf}-{\Phi }_{2}\left({\sigma }_{nf}-{\sigma }_{s2}\right)}{\left({\sigma }_{nf}-{\sigma }_{s2}\right){\Phi }_{2}+{\sigma }_{nf}+{\sigma }_{s2}}\right]\times [\frac{{\sigma }_{s1}+{\sigma }_{f}-{\Phi }_{1}\left({\sigma }_{f}-{\sigma }_{s1}\right)}{{\sigma }_{f}+{\Phi }_{1}\left({\sigma }_{f}-{\sigma }_{s1}\right)+{\sigma }_{s1}}]$
$\left({k}_{hnf}\right)$—thermal conductivity	${A}_{4}=\frac{{k}_{hnf}}{{k}_{nf}}\times \frac{{k}_{nf}}{{k}_{f}} =\frac{\left({k}_{{s}_{2}}+(\text{S}-1){k}_{nf}\right)-(\text{S}-1){\Phi }_{2}\left({k}_{nf}-{k}_{{s}_{2}}\right)}{\left({k}_{{s}_{2}}+(\text{S}-1){k}_{f}\right)+{\Phi }_{2}\left({k}_{nf}-{k}_{{s}_{2}}\right)}\times \frac{\left((\text{S}-1){k}_{f}+{k}_{{s}_{1}}\right)-(\text{S}-1){\Phi }_{1}\left({k}_{f}-{k}_{{s}_{1}}\right)}{\left((\text{S}-1){k}_{f}+{k}_{{s}_{1}}\right)+{\Phi }_{1}\left({k}_{f}-{k}_{{s}_{1}}\right)}$
${\left(\rho {c}_{p}\right)}_{hnf}$—heat capacitance	${A}_{5}=\frac{{\left(\rho {c}_{p}\right)}_{hnf}}{{\left(\rho {c}_{p}\right)}_{f}}=\left[\left(1-{\Phi }_{2}\right)\left\{{\Phi }_{1}\frac{{\left(\rho {c}_{p}\right)}_{{s}_{1}}}{{\left(\rho {c}_{p}\right)}_{f}}+\left(1-{\Phi }_{1}\right)\right\}+{\Phi }_{2}\frac{{\left(\rho {c}_{p}\right)}_{{s}_{2}}}{{\left(\rho {c}_{p}\right)}_{f}}\right]$

**Table 2:** Nanoparticles and conventional fluid thermo-physical characteristics (Nazir et al.^50^ and Khan et al.^51^).

Properties constituents	${c}_{p}$ (J kg^−1^ K^−1^)	$k$ (W mK^−1^)	$\sigma$ (Ω m^−1^)	$\beta$ (1 K^−1^)	$\rho$ (kg m^−3^)
MoS_2_	397.21	904.4	2.09 × 10^−5^	2.8424 × 10^−5^	5060
Ag	235	429	6.30 × 10^7^	1.89 × 10^−5^	10,490
H_2_O	4179	0.613	0.05	21	997.1

## Numerical method

Equations (19)–(23) with boundary conditions (24) and (25) are numerically solved in MATLAB using the well-known shooting approach with the RKF-45th method. The most effective way for computing the numerical approximation of this kind of highly nonlinear problem is the shooting method. Unlike other numerical techniques, this method lacks any complex discretization and has excellent solution accuracy. This numerical accuracy is also determined to be excellent.

32 $$u_{1} = h,u_{2} = F,u_{3} = F^{\prime},u_{3}^{\prime } = F^{\prime\prime},$$

33 $$u_{4} = \theta ,u_{5} = \theta ^{\prime},u_{5}^{\prime } = \theta ^{\prime\prime},$$

34 $$u_{6} = \phi ,u_{7} = \phi ^{\prime},u_{7}^{\prime } = \phi ^{\prime\prime},$$

35 $$u_{8} = \Theta ,u_{9} = \Theta ^{\prime},u_{9}^{\prime } = \Theta ^{\prime\prime},$$

36 $${{u}_{1}^{^{\prime}}=-2u}_{2},$$

37 $${u}_{2}^{^{\prime}}= \frac{1}{{{e}^{-{u}_{4}{\varepsilon }_{1}}A}_{1}}({A}_{2}\left( {u}_{2}^{2}+{u}_{1}{u}_{2}\right)+{u}_{2}{u}_{5}{\varepsilon }_{1}{e}^{-{u}_{4}{\varepsilon }_{1}}+{ A}_{3}M{u}_{2} +{A}_{1}{e}^{-{u}_{6}{\varepsilon }_{1}}K{u}_{2}+{F}_{r}{u}_{2}^{2}),$$

38 $${u}_{4}^{^{\prime}}={u}_{5},$$

39 $${u}_{5}^{^{\prime}}=({A}_{4}\left(1+{\varepsilon }_{2}{u}_{4}\right)+Rd-{Pr\gamma }_{1}({u}_{1}{)}^{2}{)}^{-1}({A}_{5}\left({\text{Pr}(2 {u}_{2}{u}_{4}-u}_{1}{u}_{5}\right)-\text{Ec}{A}_{1}{e}^{-{u}_{6}{\varepsilon }_{1}}({u}_{3}{)}^{2}-\text{MEc}{A}_{3}({u}_{2}{)}^{2}{+\gamma }_{1}\text{Pr}\left(4{u}_{2}^{2}{u}_{4}+4{u}_{1}{u}_{2}{u}_{5}+2{u}_{1}{u}_{3}{u}_{4}+{u}_{5}{u}_{1}{u}_{2}\right)-\frac{{A}_{4}{\left(1+{\varepsilon }_{2}{u}_{4}\right)A}_{2}}{{{e}^{-{u}_{4}{\varepsilon }_{1}}A}_{1}} \left({A}^{*}{u}_{2}+ {B}^{*}{u}_{4}\right)),$$

40 $$\begin{aligned} u_{6}^{\prime } & = u_{7} ,u_{7}^{\prime } = (1 + \varepsilon _{3} u_{6} ) - Sc\gamma _{2} (u_{1} )^{2} )^{{ - 1}} \left( {({\text{Sc}}(2u_{2} u_{6} - u_{1} u_{7} )} \right. \\ & \quad + \tau {\text{Sc}}(u_{7} u_{5} + u_{6} u_{5}^{\prime } ) - \varepsilon _{3} (u_{7} )^{2} + RcSc\exp \left( {\frac{{ - E}}{{1 + \delta u_{4} }}} \right)(1 + \delta u_{4} )^{m} u_{6} + \gamma _{1} \Pr \left( {4u_{2}^{2} u_{6} } \right. \\ & \left. {\left. {\quad + 4u_{1} u_{2} u_{7} + 2u_{1} u_{3} u_{6} + u_{7} u_{1} u_{2} } \right)} \right), \\ & u_{8}^{\prime } = u_{9} ,u_{9}^{\prime } = (({\text{Sb}}(2u_{2} u_{8} + u_{1} u_{9} ) + Pe(u_{7} u_{9} + ({{\upsigma }} + u_{8} )u_{7}^{\prime } ). \\ \end{aligned}$$

Boundary conditions

41 $${u}_{1}\left(0\right)=0, {u}_{2}\left(0\right)={n}_{1}, {u}_{3}\left(0\right)=-2\frac{Mn\left(1+Ma\right)}{{{e}^{-{u}_{4}{\varepsilon }_{1}}A}_{1}},$$

42 $${u}_{4}\left(0\right)=1,{u}_{5}\left(0\right)={n}_{2},$$

43 $${u}_{6}\left(0\right)=1, {u}_{7}\left(0\right)={n}_{3},$$

44 $${u}_{8}\left(0\right)=1, {u}_{9}\left(0\right)={n}_{4}.$$

**Figure 6:**
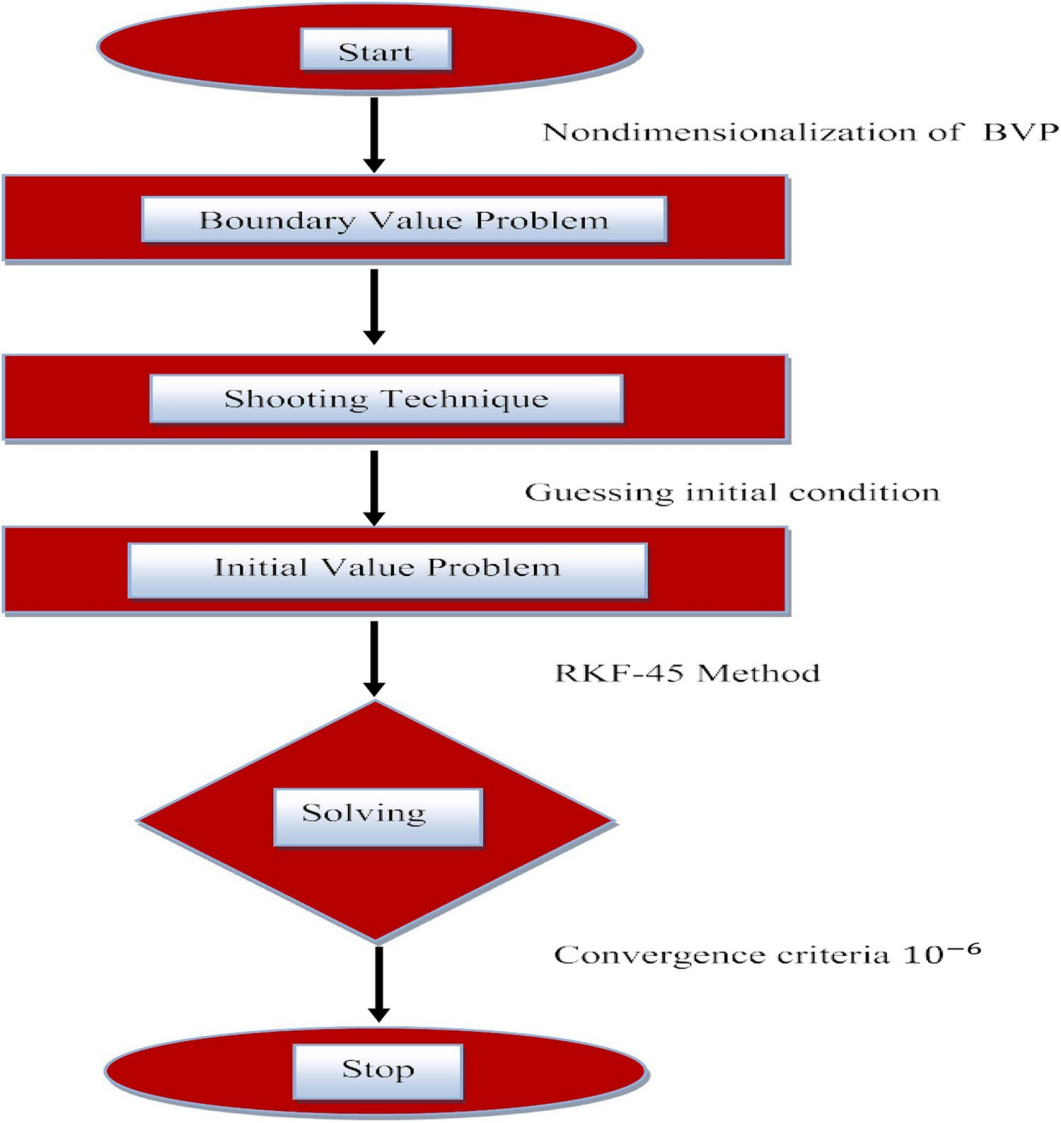
Solution procedure.

The unknowns ${n}_{1}$ to ${n}_{4}$ are estimated using the shooting method. The norms for convergence are ${10}^{-6}$ and the numerical corroboration is used, with a maximum step size of 0.001. The solution procedure of the problem is given in Fig. 6.

## Results and discussions

**Figure 7:**
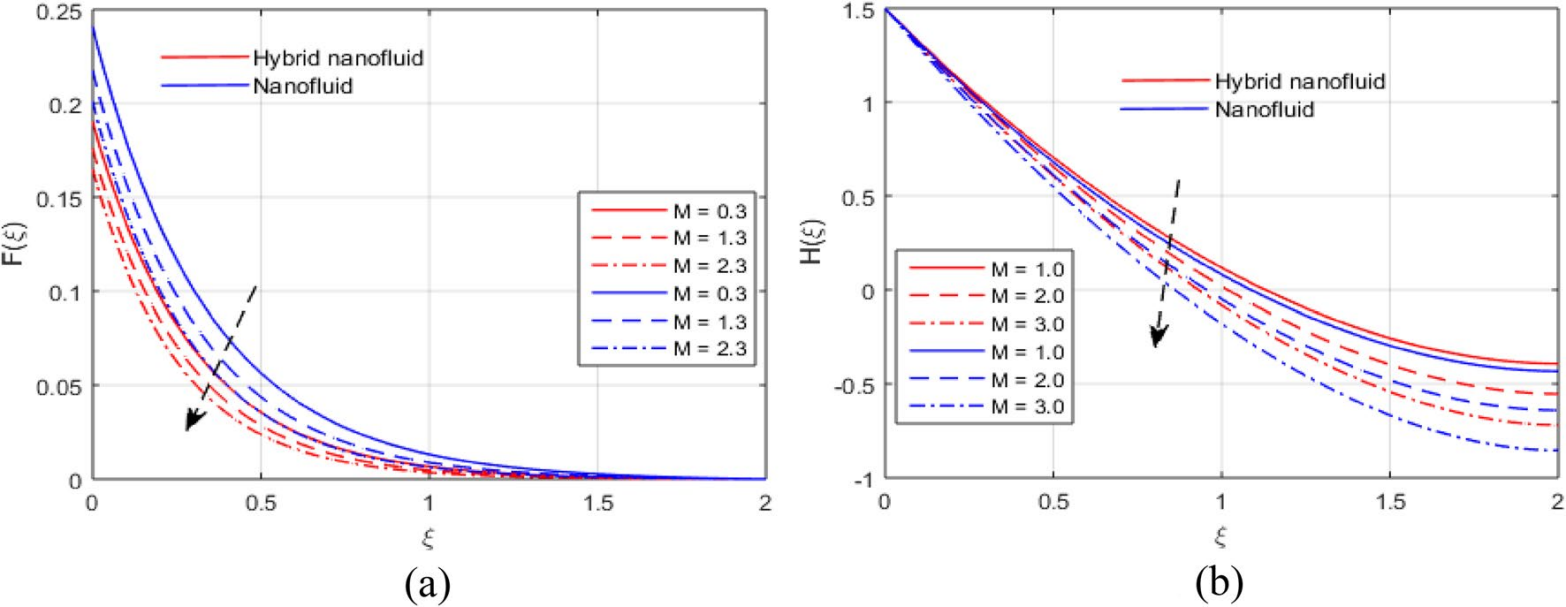
(**a**, **b**) Influence of $M$ on $F\left(\xi \right)$ and $H\left(\xi \right)$.

**Figure 8:**
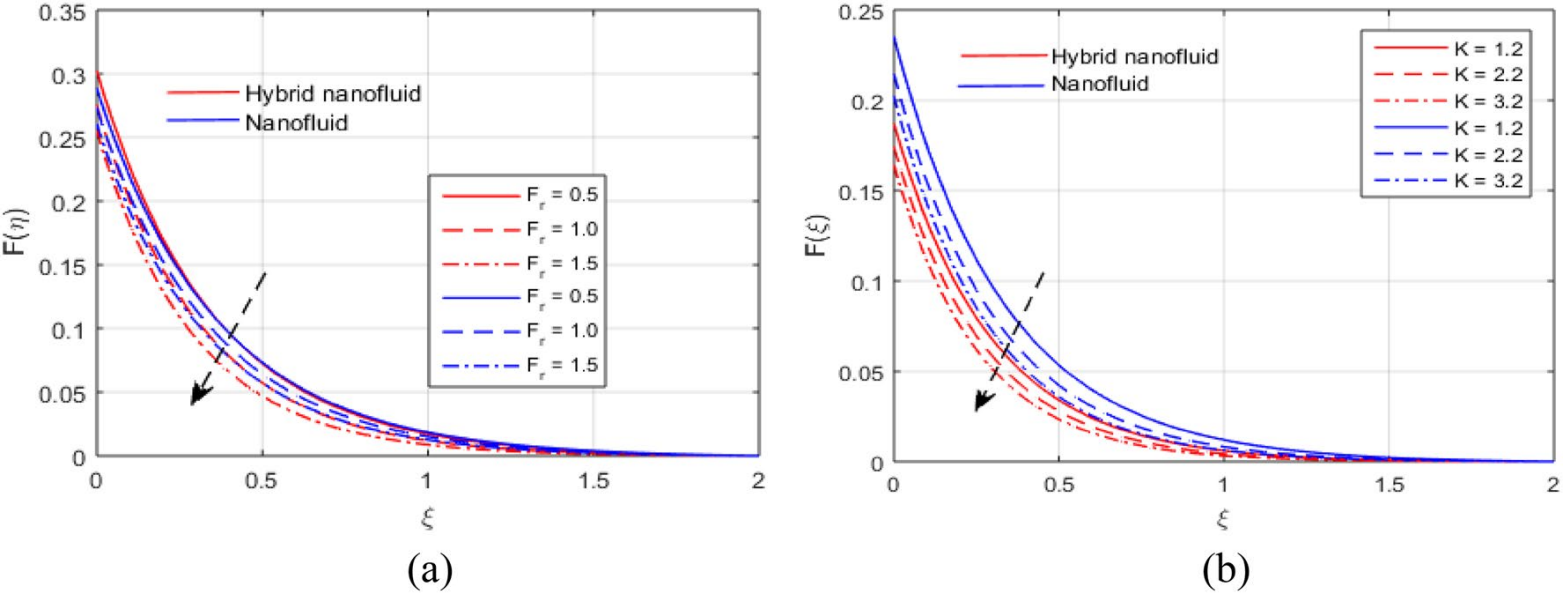
(**a**, **b**) Effect of ${F}_{r}$ and $K$ on $F\left(\xi \right)$.

**Figure 9:**
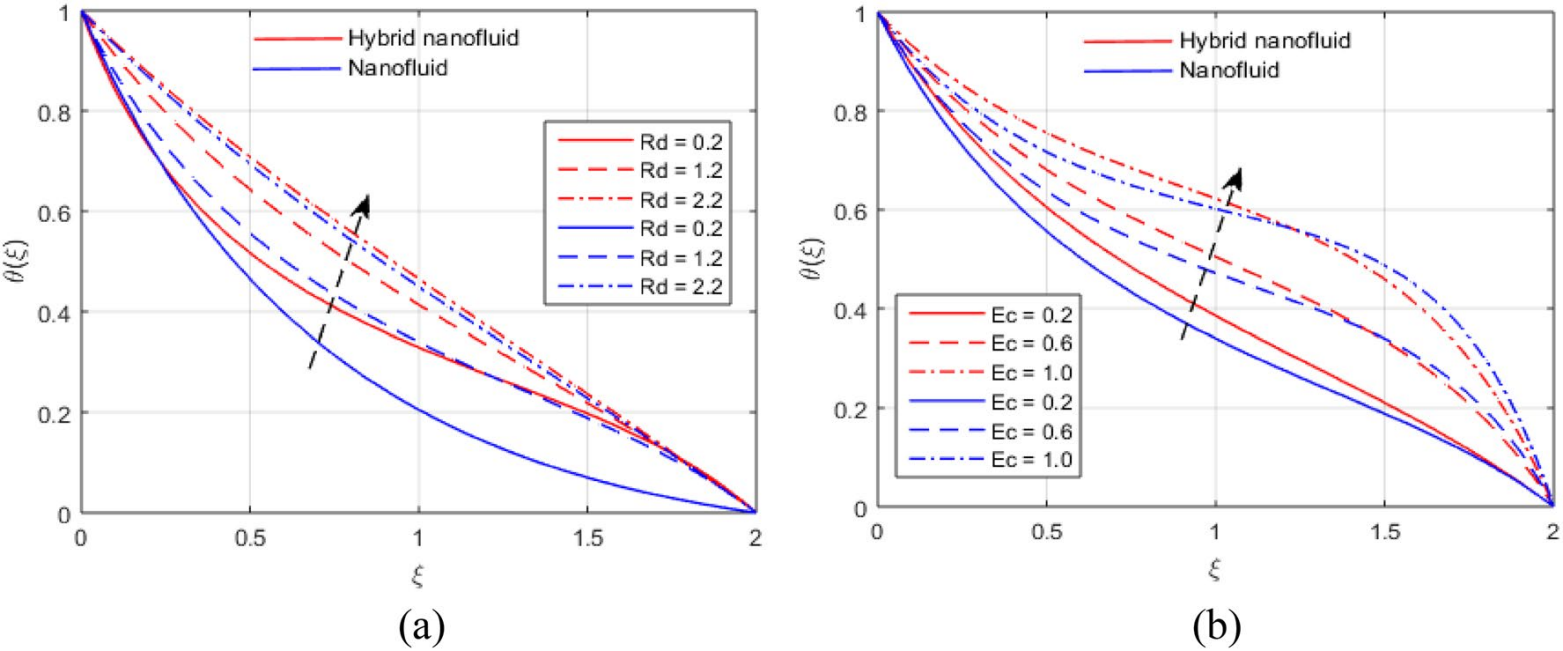
(**a**, **b**) Influence of $Rd$ and $Ec$ on $\theta \left(\xi \right)$.

**Figure 10:**
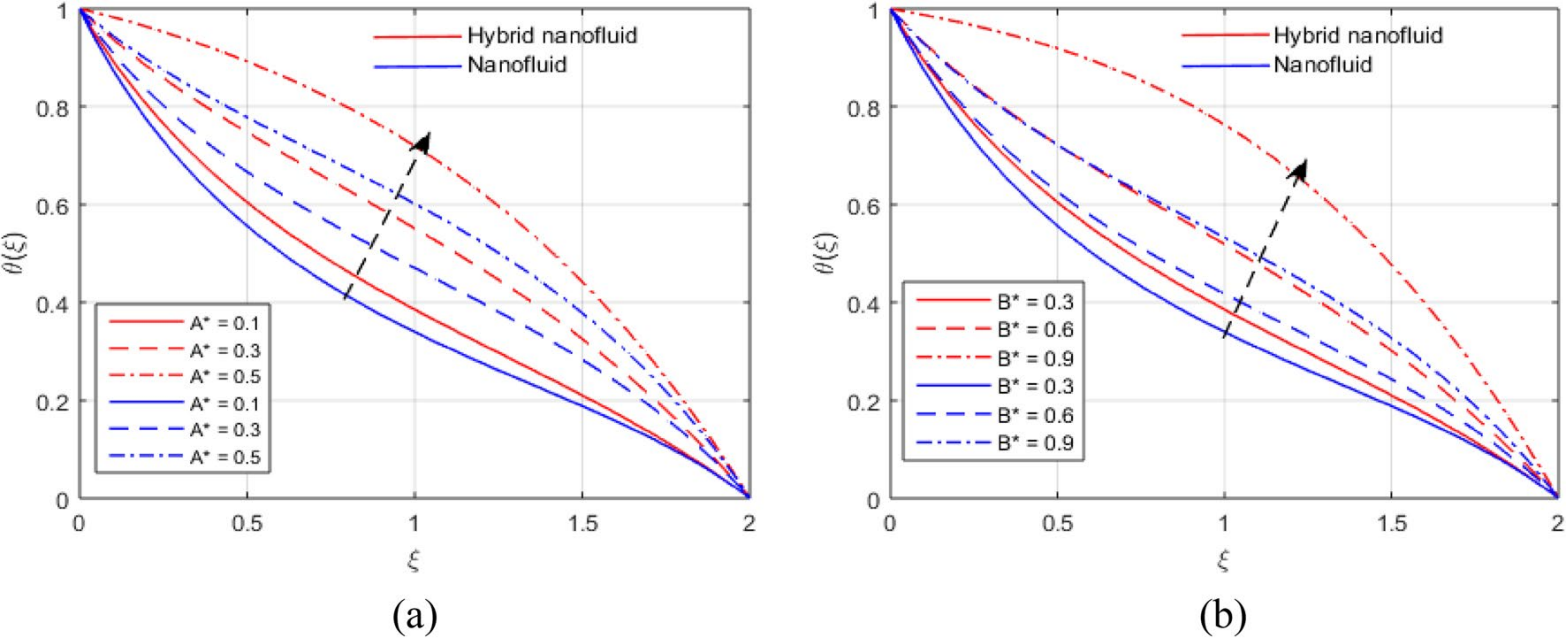
(**a**, **b**) Effect of ${A}^{*}$ and ${B}^{*}$ on $\theta \left(\xi \right)$.

**Figure 11:**
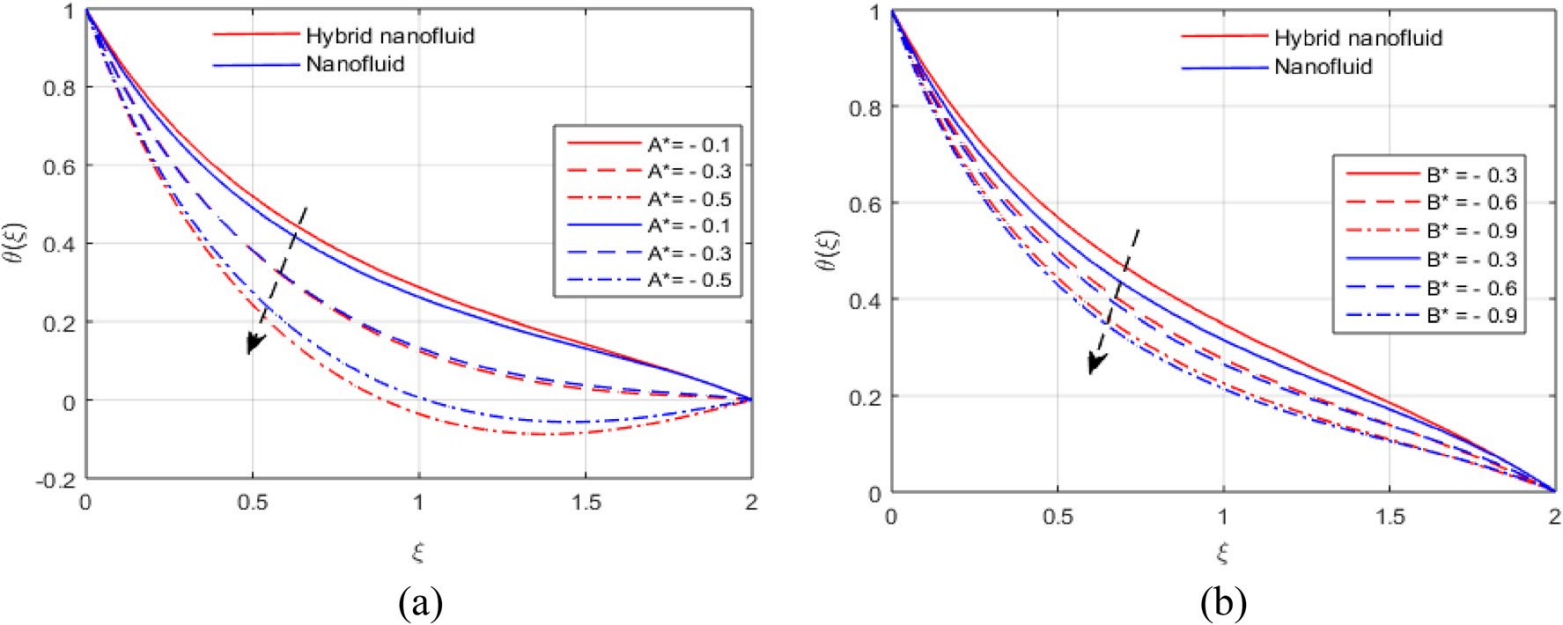
(**a**, **b**) Influence of ${A}^{*}$ and ${B}^{*}$ on $\theta \left(\xi \right)$.

**Figure 12:**
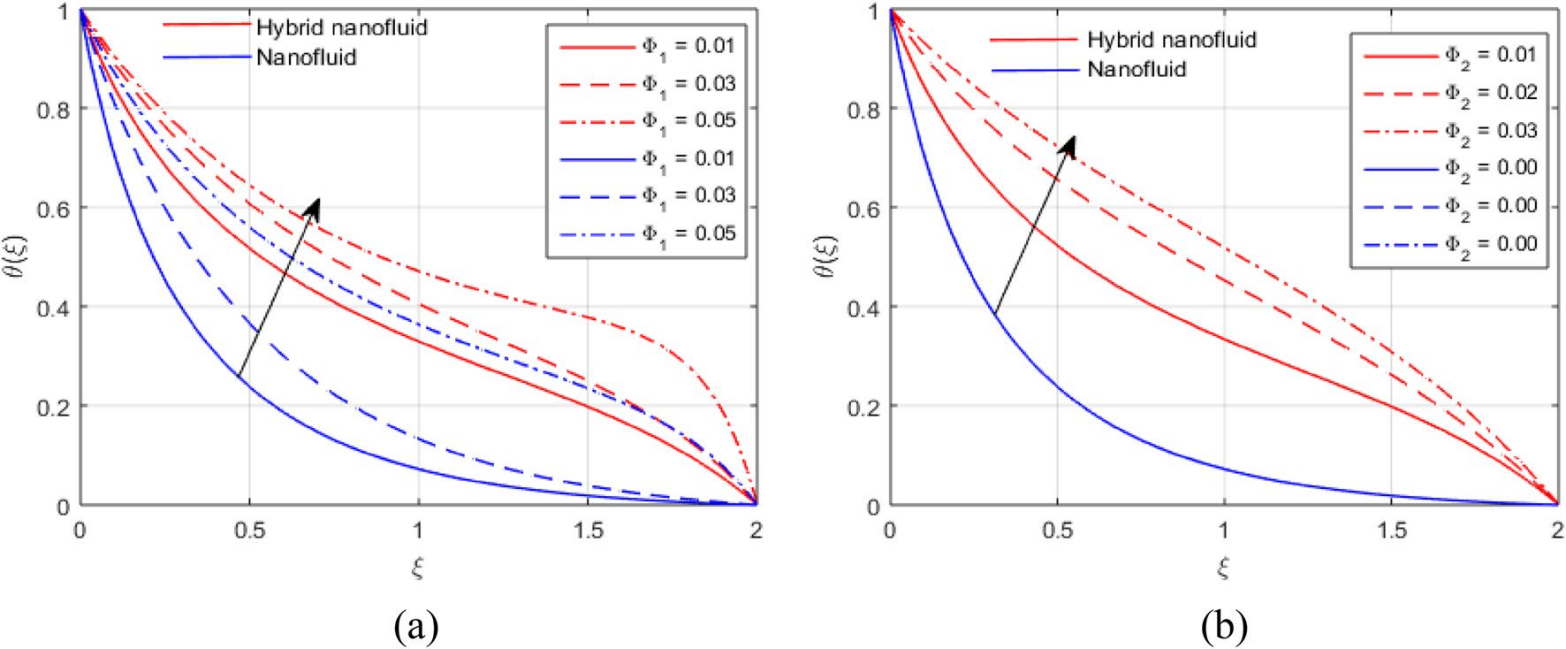
(**a**, **b**) Effect of ${\Phi }_{1}$ and ${\Phi }_{2}$ and $\theta \left(\xi \right)$.

**Figure 13:**
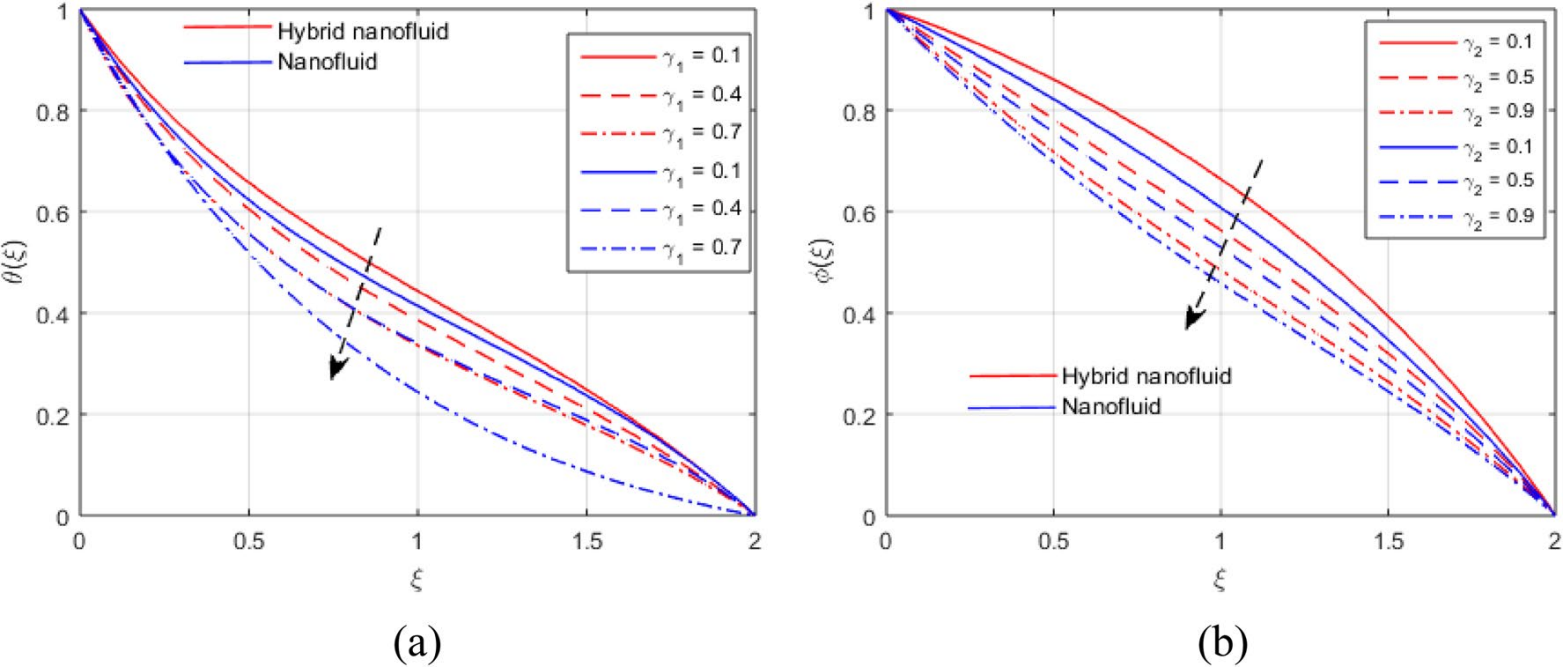
(**a**, **b**) Effect of ${\gamma }_{1}$ and ${\gamma }_{2}$ on $\theta \left(\xi \right)$ and $\phi \left(\xi \right)$.

**Figure 14:**
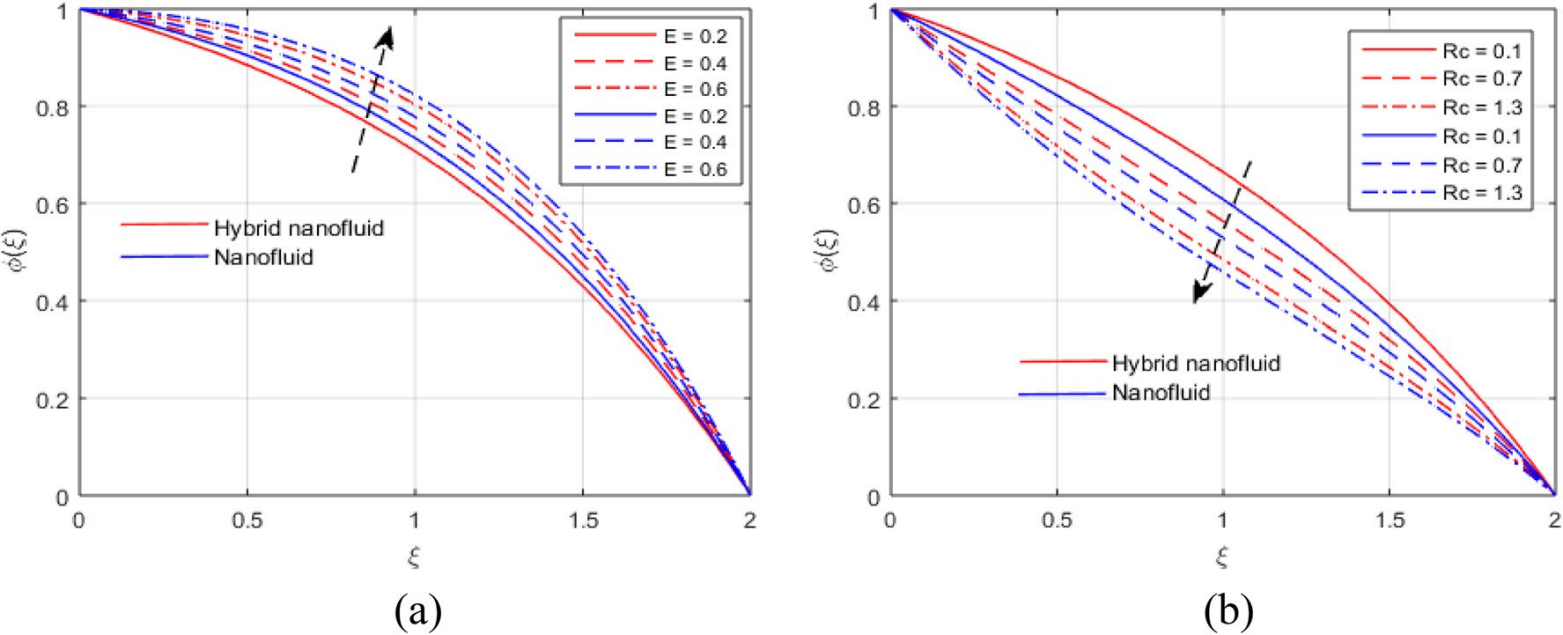
(**a**, **b**) Effect of $E$ and $Rc$ on $\phi \left(\xi \right)$.

**Figure 15:**
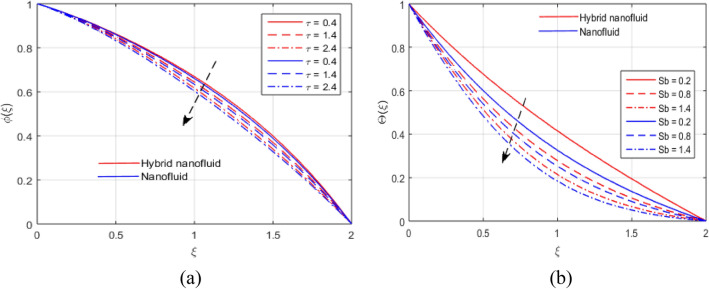
(**a**, **b**) Effect of $\tau$ and $Lb$ on $\phi \left(\xi \right)$ and $\Theta \left(\xi \right)$.

**Figure 16:**
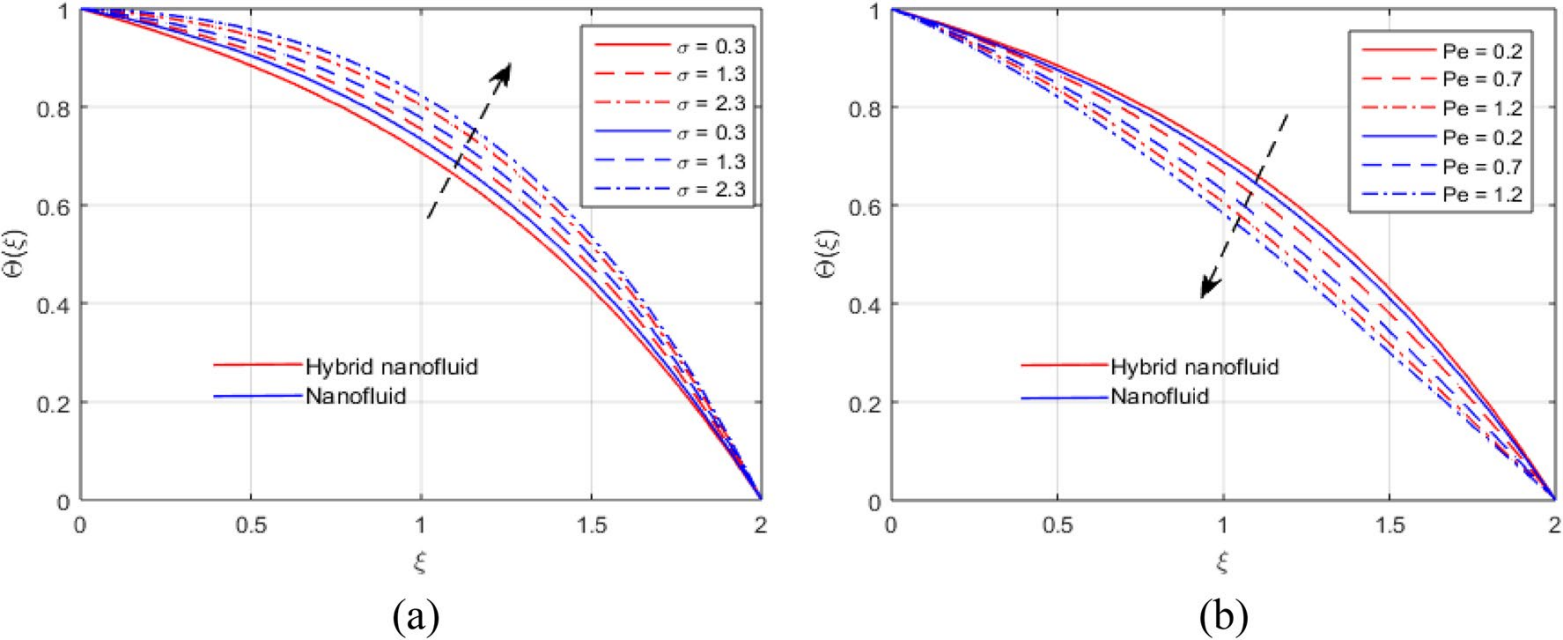
(**a**, **b**) Effect of $\sigma$ and $Pe$ on $\Theta \left(\xi \right)$.

**Figure 17:**
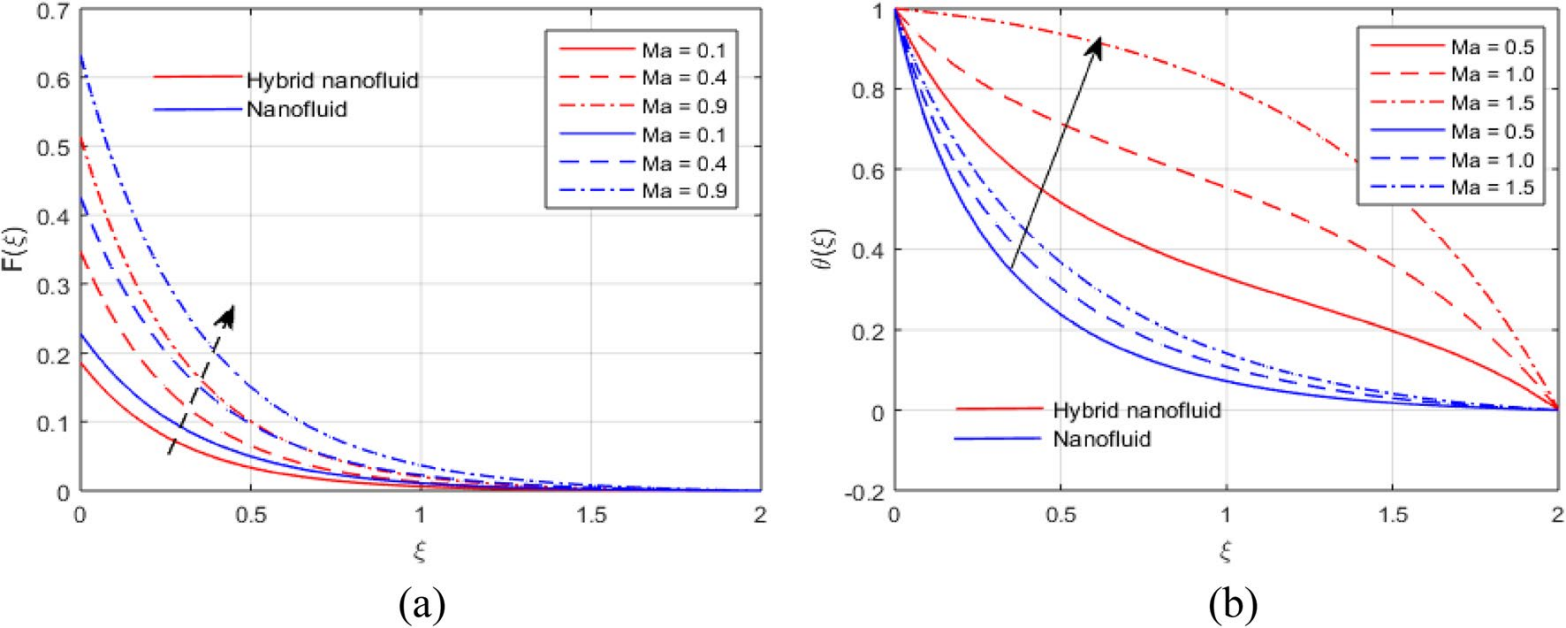
(**a**, **b**) Influence of $Ma$ on $F\left(\xi \right)$ and $\theta \left(\xi \right)$.

**Figure 18:**
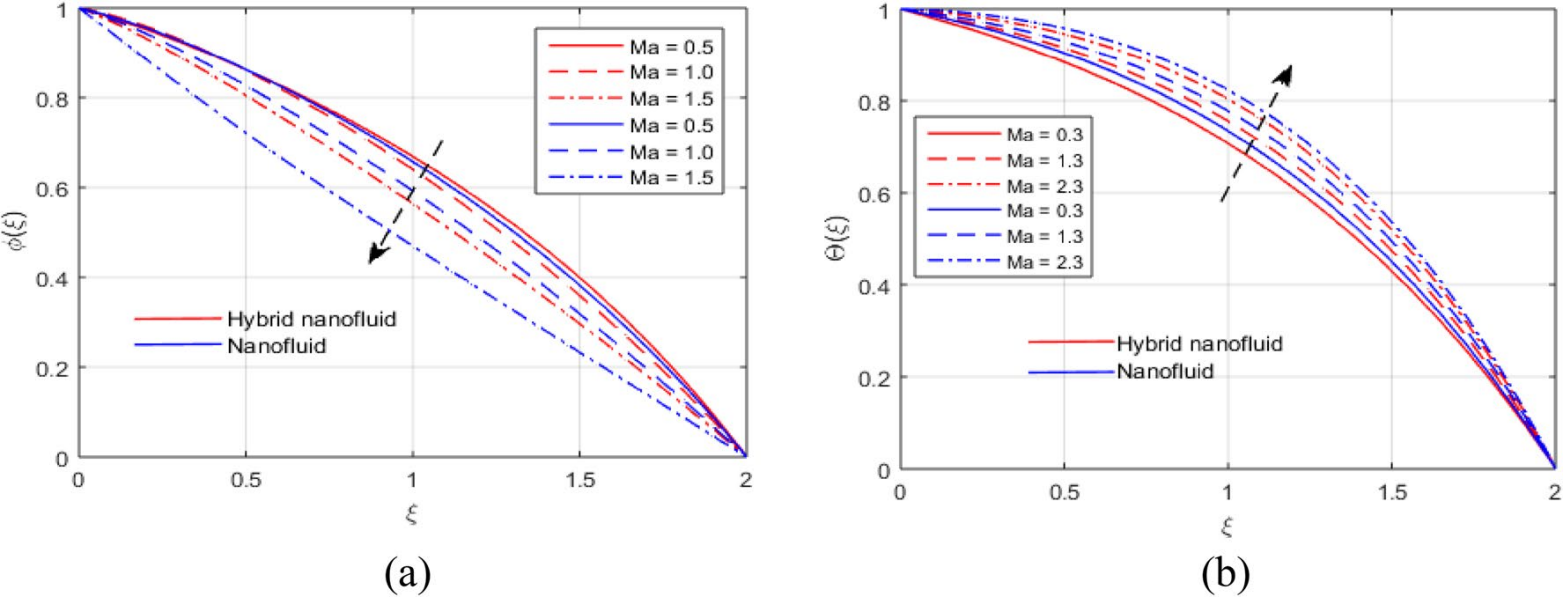
(**a**, **b**) Effect of $Ma$ on $\phi \left(\xi \right)$ and $\Theta \left(\xi \right)$.

**Figure 19:**
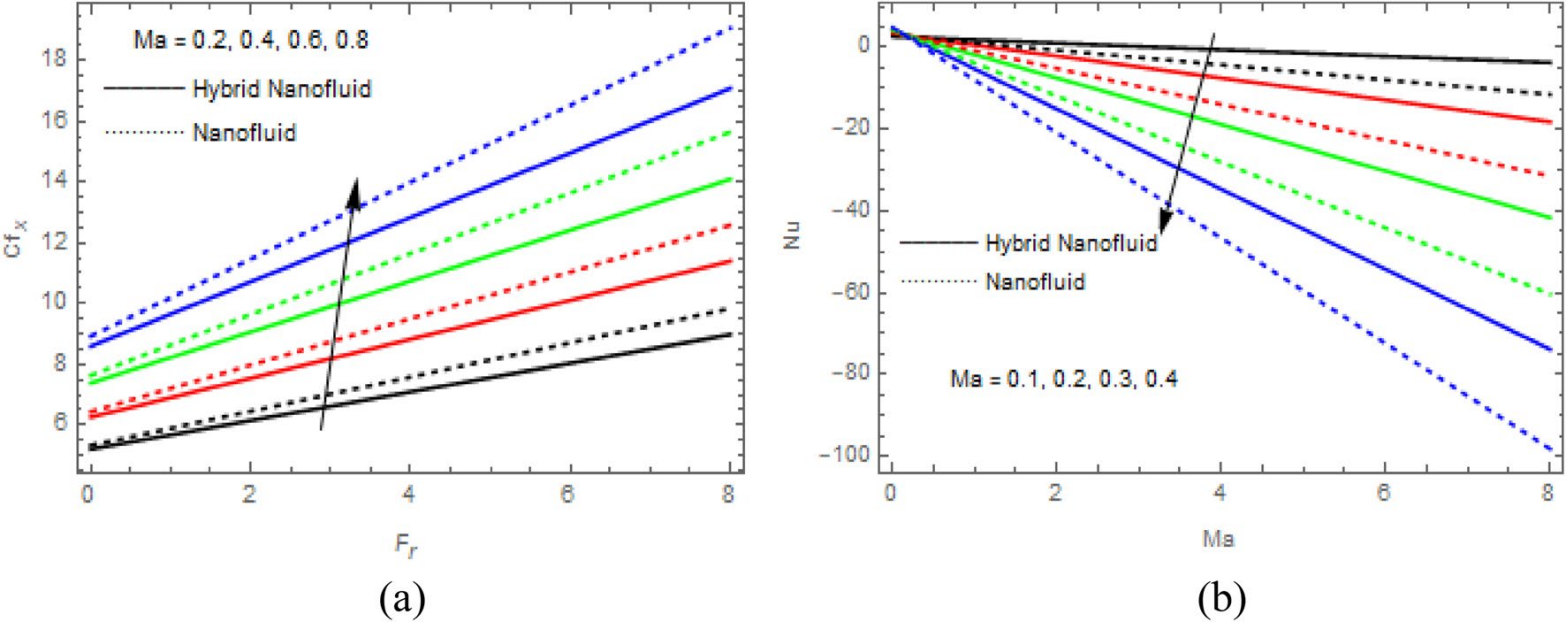
(**a**, **b**) Influence of $Ma$ on ${Cf}_{x}$ and $Nu$.

**Figure 20:**
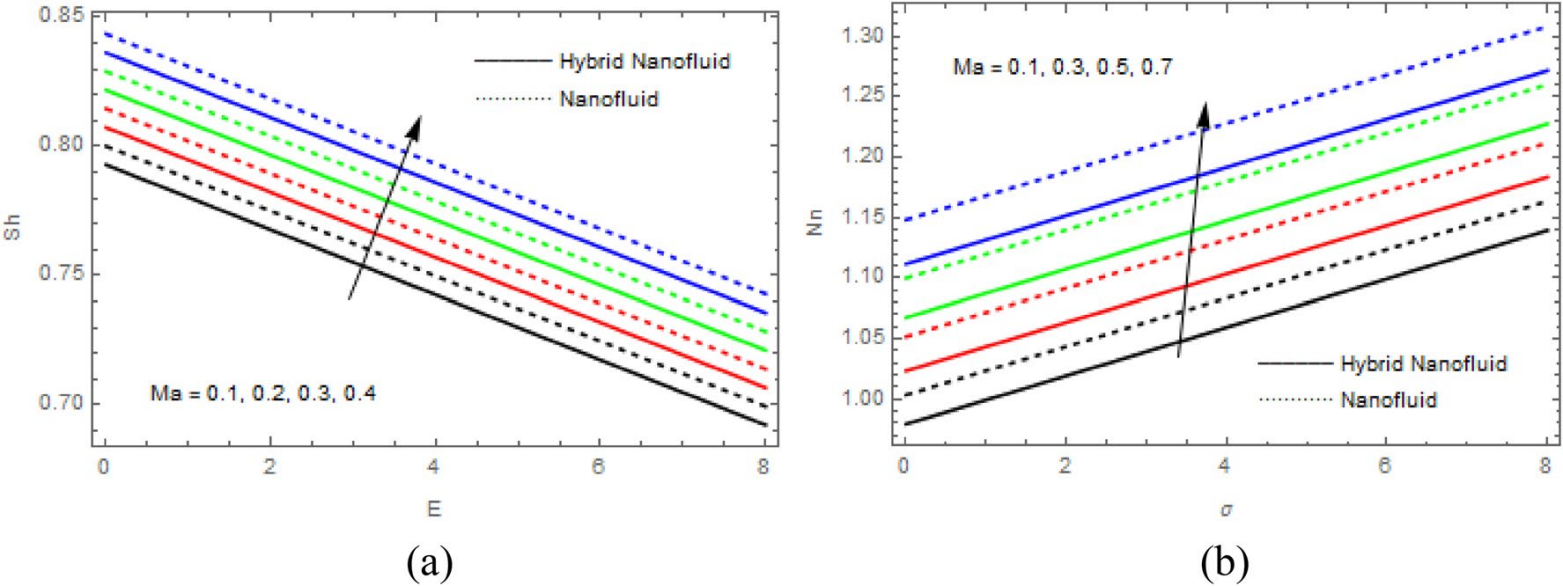
(**a**, **b**) Influence of $Ma$ on $Sh$ and $Nn$.

The presumed flow modeled equations are transformed to ODEs by selecting the appropriate similarity variables. The primary goal of this section is the physical explanation of involved parameters for discrete flow fields. Figures 7, 8, 9, 10, 11, 12, 13, 14, 15, 16, 17 and 18 have been arranged and plotted. Table 2 displays the thermo-physical features of carrier liquid and nanoparticles. The impact of dimensionless parameters such as Marangoni convection parameter, inverse Darcy number, Prandtl number, Schmidt number, Forchheimer parameter, temperature difference parameter, chemical reaction parameter, concentration difference parameter, reaction rate co-efficient, the reference length and the activation energy are discussed. The impact of $M$ on $F\left(\xi \right)$ and $H\left(\xi \right)$ is seen in Fig. 7a,b$.$ The velocity profiles drop as a result of an rise in the values of $M$. The transverse magnetic field application will produce a resistive force similar to the drag force, which has the tendency to slow down the velocity of the hybrid nanofluid. Figure 8a,b demonstrate the outcome of ${F}_{r}$ and $K$ on $F\left(\xi \right)$. The velocity gradient declines as a result of the growth in $K$ and ${F}_{r}$ values, as shown in the graph. The plot shows that hybrid nanofluid velocity and associated boundary thickness decreases as $K$ and ${F}_{r}$ increases. This is because an increase in porosity widens the pores in a porous medium, which causes resistive forces to operate against flow and lower velocity profiles. The impact of $Ec$ and $Rd$ on $\theta \left(\xi \right)$ are shown in Fig. 9a,b$.$ When the values of $Ec$ are raised, the temperature rises. The Eckert number $Ec$ describes the relationship between the enthalpy and the flow's kinetic energy. It represents the process by which kinetic energy is transformed into internal energy by effort against viscous fluid forces. The temperature of nanofluid and hybrid nanofluid increases due to the increased viscous dissipative heat. By raising $Rd$, the temperature and the boundary layer thickness that corresponds to it rise. When we use the impacts of thermal radiation, the surface heat flux physically increases, and this becomes the key to raising temperature. The impact of ${A}^{*}$ and ${B}^{*}$ on $\theta \left(\xi \right)$ is depicted in Figs. 10a,b and 11a,b. It is found that improving ${A}^{*}$ and ${B}^{*}$ values result in better temperature distributions for hybrid nanofluid and nanofluid. The non-uniform heat sources ${A}^{*}$ and ${B}^{*}$ are considered heat sources when they release heat energy into the fluid flow and operate as heat generators, which causes the temperature distribution to become more uniform. However, they are referred to as heat sinks when the non-uniform heat sources/sinks ${A}^{*}$ and ${B}^{*}$ are given negative values. The boundary layer's function as a heat sink reduces the temperature of the hybrid and nanofluid nanofluids. Figure 12a,b show how the temperature gradient changes for increasing volume fractions. The temperature distribution and thickness of the associated boundary layer both grow as the volume fractions ${\Phi }_{1}$ and ${\Phi }_{2}$ rise. Additionally, as the volume percentage of nanoparticles rises, more heat is produced, which improves the thermal profile and increases the thickness of the associated boundary layer. The impacts of ${\gamma }_{1}$ and ${\gamma }_{2}$ on $\theta \left(\eta \right)$ and $\phi \left(\eta \right)$ are described in Fig. 13a,b. The thermal $\theta \left(\xi \right)$ and concentration $\phi \left(\xi \right)$ profiles tend to decline with the increasing relaxation times, as demonstrate by careful examination of the aforementioned figures. These figures also show that the concentration and thermal boundary layer thicknesses for the traditional Fourier's law and Fick's law when mass and heat quickly pass throughout the material (i.e., ${\gamma }_{1}={\gamma }_{2}=0$) are greater than for the Cattaneo-Christov double-diffusion model. Figure 14a,b show the bearing of the solutal profile for various values of $E$ and $Rc$. The concentration gradient is enhanced by the increase in $E$ values, whereas the trend is the opposite for increasing $Rc$ values, as shown in Fig. 14a,b$.$ Stronger chemical reactions have a destructive outcome that causes the reactant species to deteriorate. Figure 15a illustrates how the influence of $\tau$ is on $\phi \left(\xi \right).$ The solutal profile $\phi \left(\xi \right)$ drops as the values of $\tau$ rise, as seen in Fig. 15a$.$ When the temperature gradient grew, a weaker concentration is seen because of an growth in particle mobility. The outcome of $Lb$ on $\Theta \left(\xi \right)$ is depicted in Fig. 15b. It is clear that when $Sb$ increases, the density profile of mobile organisms in hybrid and nanofluid diminish. The impact of $\sigma$ and $Pe$ on microorganism profile is depicted in Fig. 16a,b$.$ Both the hybrid nanofluid and nanofluid microorganism profiles dropped when we enhanced $Pe$ and $\sigma$ values. Inversely proportional to ${D}_{m}$ (microorganisms diffusivity) and directly proportional to one another are the Peclet number $(Pe)$ and cell swimming speed (${W}_{c}$). Advection and diffusion rates are related to the Peclet number. As a result, a rise in $Pe$ results from an increase in the rate of advective transport, this quickly increases the flux of microorganisms. The impact of $Ma$ on $F\left(\xi \right),\theta \left(\xi \right),\phi \left(\xi \right)$ and $\Theta \left(\xi \right)$ are depicted in Figs. 17a,b and 18a,b, respectively. The graphs illustrate how raising $Ma$ values enhance the microorganism, temperature and velocity profiles of hybrid and nanofluid. This phenomenon is brought on by the surface variation. A stronger Marangoni effect will almost always lead to a higher velocity gradient because it works as a pouring force for liquid streams. These graphs show that as the value of $Ma$ increases, the concentration $\phi \left(\xi \right)$ profile significantly decreases. The Marangoni number is physically connected to the surface tension. The bulk attraction of the liquid to the particles in the surface layer is which creates surface tension on a liquid's surface and because of this, as surface tension rises, surface molecule attraction grows stronger. Figures 19a,b and 20a,b show how $Ma$ affects the Nusselt number $(Nu)$, local density microorganisms $(Nn)$, Sherwood number $(Sh)$ and skin friction $(C{f}_{x})$. Skin friction, local density microorganisms and Sherwood number are improved as $Ma$ increases, whereas Nusselt number declines. Table 3 shows the impact of several parameters on Nusselt number $(Nu)$. Table 4 displays the influence of numerous parameters on Sherwood number $(Sh)$. Table 5 shows the effect of several parameters on local density of motile microorganisms $(Nn)$. Table 6 shows the shape factors of nanoparticle. Table 7 displays the comparison results of the present study to earlier published research, with the additional parameters set to zero.

**Table 3:** Impact of several parameters on Nusselt number.

$Ma$	$Rd$	${A}^{*}$	${B}^{*}$	${\gamma }_{1}$	$Ec$	${\Phi }_{1}$	${\Phi }_{2}$	${\upepsilon }_{2}$	$M$	$\mathrm{Nu}=-{ (\mathrm{Re})}^{1/2}\left[{A}_{5}\left(1+{\epsilon }_{2}\theta \left(0\right)\right)+\mathrm{Rd}\right]{\theta }^{\mathrm{^{\prime}}}\left(0\right)$	
										Hybrid nanofluid ${\mathrm{MoS}}_{2}+\mathrm{ Ag}-{H}_{2}O$	Nanofluid ${\mathrm{MoS}}_{2}-{H}_{2}O$
**0.2**	$0.3$	$0.4$	$0.1$	$0.6$	$0.7$	$0.03$	$0.04$	$0.4$	$0.5$	$2.771923$	$2.518590$
**0.3**										$2.769145$	$2.504640$
**0.4**										$2.762259$	$4.932424$
	**0.3**									$2.185224$	$1.936823$
$0.4$	**0.4**	$0.3$	$0.1$	$0.6$	$0.7$	$0.03$	$0.04$	$0.4$	$0.5$	$2.478792$	$2.481982$
	**0.5**									$2.771923$	$2.518590$
		**0.2**								$2.771923$	$2.518590$
$0.1$	$2.0$	**0.3**	$0.1$	$0.6$	$0.7$	$0.03$	$0.04$	$0.4$	$0.5$	$2.741044$	$2.504221$
		**0.4**								$2.718525$	$2.495601$
			**0.2**							$2.771923$	$2.468590$
$0.2$	$2.0$	$0.3$	**0.3**	$0.6$	$0.7$	$0.03$	$0.04$	$0.4$	$0.5$	$2.767468$	$2.441035$
			**0.4**							$2.737217$	$2.427719$
				**0.2**						$2.758276$	$2.505453$
$0.2$	$2.0$	$0.3$	$0.1$	**0.3**	$0.7$	$0.03$	$0.04$	$0.4$	$0.5$	$2.762491$	$2.514638$
				**0.4**						$2.766963$	$2.523625$
					**0.2**					$2.766963$	$2.503625$
$0.2$	$2.0$	$0.3$	$0.1$	$0.6$	**0.3**	$0.03$	$0.04$	$0.4$	$0.5$	$2.767111$	$2.489524$
					**0.4**					$2.767252$	$2.463933$
						**0.02**				$2.766232$	$2.512736$
$0.2$	$2.0$	$0.3$	$0.1$	$0.6$	$0.7$	**0.04**	$0.04$	$0.4$	$0.5$	$3.052429$	$2.760667$
						**0.06**				$3.370178$	$3.036003$
							**0.01**			$2.766232$	–
$0.2$	$2.0$	$0.3$	$0.1$	$0.6$	$0.7$	$0.03$	**0.02**	$0.4$	$0.5$	$3.046353$	–
							**0.03**			$3.356580$	–
								**0.2**		$2.766232$	$2.512736$
$0.2$	$2.0$	$0.3$	$0.1$	$0.6$	$0.7$	$0.03$	$0.04$	**0.4**	$0.5$	$3.243972$	$2.943242$
								**0.6**		$4.368021$	$3.884085$
									**0.1**	$2.767670$	$2.514506$
									**0.2**	$2.766963$	$2.769360$
$0.2$	$2.0$	$0.3$	$0.1$	$0.6$	$0.7$	$0.03$	$0.04$	$0.4$	**0.3**	$2.766232$	$2.512736$

Significant values are in [bold].

**Table 4:** Influence of numerous parameters on Sherwood number.

$Ma$	$Rc$	$\tau$	${\gamma }_{2}$	${\epsilon }_{3}$	${\Phi }_{1}$	${\Phi }_{2}$	$\mathrm{Sh}=-{ (\mathrm{Re})}^{1/2}\left(1+{\epsilon }_{3}\phi \left(0\right)\right){\phi }^{\mathrm{^{\prime}}}\left(0\right)$	
							Hybrid nanofluid ${\mathrm{MoS}}_{2}+\mathrm{ Ag}-{H}_{2}O$	Nanofluid ${\mathrm{MoS}}_{2}-{H}_{2}O$
**0.1**	$0.3$	$0.4$	$0.1$	$0.6$	$0.05$	$0.04$	$1.515392$	$1.509413$
**0.3**							$1.532564$	$1.518774$
**0.5**							$1.553520$	$1.522746$
	**0.3**						$2.741115$	$1.519413$
$0.2$	**0.6**	$0.3$	$0.1$	$0.6$	$0.05$	$0.04$	$1.515392$	$0.519413$
	**0.9**						$0.515372$	$1.519213$
		**0.2**					$3.515392$	$2.519413$
$0.2$	$2.0$	**0.3**	$0.1$	$0.6$	$0.05$	$0.04$	$1.520348$	$1.522030$
		**0.4**					$1.564230$	$1.565958$
			**0.1**				$1.515392$	$1.519413$
$0.2$	$2.0$	$0.3$	**0.3**	$0.6$	$0.05$	$0.04$	$1.527311$	$1.524395$
			**0.4**				$1.534871$	$1.534076$
				**0.2**			$1.398473$	$1.669138$
$0.2$	$2.0$	$0.3$	$0.1$	**0.4**	$0.05$	$0.04$	$2.088955$	$2.096924$
				**0.6**			$2.516428$	$2.665932$
					**0.02**		$1.515392$	$1.519413$
$0.2$	$2.0$	$0.3$	$0.1$	$0.6$	**0.04**	$0.04$	$1.538053$	$1.535533$
					**0.06**		$1.557465$	$1.556503$
						**0.01**	$1.515392$	–
						**0.03**	$1.509902$	–
$0.2$	$2.0$	$0.3$	$0.1$	$0.6$	$0.05$	**0.05**	$1.454627$	–

Significant values are in [bold].

**Table 5:** Effect of several parameters on local density of motile microorganisms.

$Ma$	$Mn$	$Sb$	$\upsigma$	$Pe$	${\Phi }_{1}$	${\Phi }_{2}$	$\mathrm{Nn}=-{ \left(\mathrm{Re}\right)}^\frac{1}{2}\Theta (\mathrm{o})$	
							Hybrid nanofluid ${\mathrm{MoS}}_{2}+\mathrm{ Ag}-{H}_{2}O$	Nanofluid ${\mathrm{MoS}}_{2}-{H}_{2}O$
**0.1**	$0.3$	$0.4$	$0.1$	$0.6$	$0.05$	$0.04$	$1.457885$	$1.407926$
**0.3**							$1.475646$	$1.426974$
**0.5**							$1.494740$	$1.444515$
	**0.3**						$1.419923$	$1.426241$
$0.2$	**0.6**	$0.3$	$0.1$	$0.6$	$0.05$	$0.04$	$1.428360$	$1.449852$
	**0.9**						$2.739832$	$1.450012$
		**0.2**					$1.419923$	$1.426241$
$0.2$	$2.0$	**0.3**	$0.1$	$0.6$	$0.05$	$0.04$	$1.409923$	$1.416961$
		**0.4**					$1.299923$	$1.406660$
			**0.1**				$1.449913$	$1.426241$
$0.2$	$2.0$	$0.3$	**0.3**	$0.6$	$0.05$	$0.04$	$1.439823$	$1.406961$
			**0.4**				$1.429733$	$1.296660$
				**0.2**			$1.419923$	$1.426241$
$0.2$	$2.0$	$0.3$	$0.1$	**0.4**	$0.05$	$0.04$	$1.428913$	$1.468382$
				**0.6**			$1.431250$	$1.500491$
					**0.02**		$1.419923$	$1.426241$
$0.2$	$2.0$	$0.3$	$0.1$	$0.6$	**0.04**	$0.04$	$1.377895$	$1.389349$
					**0.06**		$1.284937$	$1.283476$
						**0.01**	$1.419923$	–
						**0.03**	$1.411479$	–
$0.2$	$2.0$	$0.3$	$0.1$	$0.6$	$0.05$	**0.05**	$1.319759$	–

Significant values are in [bold].

**Table 6:**
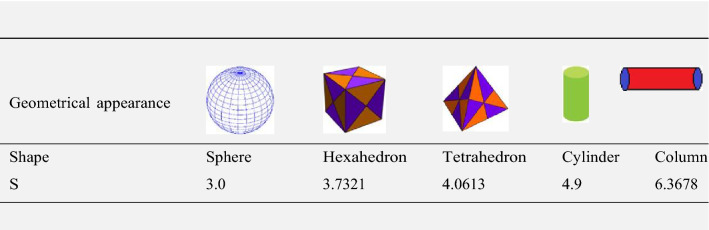
Shape factors of nanoparticles.

**Table 7:** The comparison results of the present study to earlier published research, with the additional parameters set to zero.

$Pr$	$Rd$	Nusselt number $(\mathrm{Nu})$	
		Present results	Shafiq et al. ^52^
**4.0**		$1.764114$	$1.764112$
**5.0**		$1.767002$	$1.767000$
**6.0**		$1.768492$	$1.768490$
	**0.0**	$1.488654$	$1.488649$
	**0.1**	$1.551385$	$1.551383$
	**0.2**	$1.609966$	$1.609960$

Significant values are in [bold].

## Concluding remarks

Numerical analysis is done to determine the importance of the thermo-solutal Marangoni 1convective flow of hybrid fluid across an infinite disc containing thermopherotic patricles, microorganisms and activation energy. The thermal energy analysis makes use of the Cattaneo-Christov model. Some important conclusions are drawn from this research.

- The solutal and thermal layer thicknesses are increased but the velocity is decreased by the Lorentzian body strength. This is caused by the magnetic field's imposed retardation force.
- Higher values of the Darcy number and the Forchheimer parameter results in a reduction in the axial velocity profile.
- The temperature field is significantly improved by thermal energy modulations (Space-dependent coefficient and Temperature coefficient), which both add more heat to the hybrid nanoliquid system.
- Increases in the Marangoni ratio parameter, chemical reaction parameter, thermophoretic parameter and concentration relaxation time cause the hybrid nanofluid concentration profile to fall, whereas the activation energy parameter exhibits the opposite behavior.
- The hybrid nanofluids velocity, microorganisms and temperature increase due to Marangoni convection.
- The Nusselt number shows increasing behavior by increasing the solid volume fractions.
- By raising the chemical reaction parameter, and thermopherotic parameter, Sherwood number significantly decreased.
- By raising the Schmidt number for bio-convection, the local density of motile microorganisms gets declined.
- The calculations show that our results are in good accord with the previous research.

## Data Availability

All data generated or analysed during this study are included in this published article.

## List of symbols

$(r, \phi , z)$: Cylindrical coordinate system
$\left(u w\right)$: Velocity fields of fluid (m s^−1^)
$C$: Species of the fluid concentration (m)
$N$: Density of motile microorganism
${D}_{B}$: Mass diffusivity coefficient (m^2^ s^−1^)
${W}_{c}$: Maximum cell swimming speed
${D}_{m}$: Diffusivity of microorganisms
${K}^{*}$: Chemical reaction co-efficient (mol L^−1^ s^−1^)
${k}_{1}$: Permeability of the porous medium (m^2^)
$$k$$: Thermophoretic coefficient
$${T}_{\text{f}}$$: Reference temperature (k)
${C}_{p}$: Specific heat of the fluid (J kg^−1^ k^−1^)
$${T}_{0}$$: Constants
$$T$$: Fluid temperature (k)
$$Sc$$: Schmidt number
$$Ec$$: Eckert number
${k}_{f}$: Thermal conductivity of the fluid (W m^−1^ k^−1^)
$${F}_{r}$$: Forchheimer parameter
${T}_{\infty }$: Fluid ambient temperature (k)
B*: Temperature coefficient (k)
$$Pr$$: Prandtl number
$E$: Activation energy parameter
Pe: Bioconvection Peclet number
${B}_{0}$: Uniform magnetic field (kg s^−2^ A^−1^)
q: Normal heat flux (W m^−2^)
J: Normal mass flux (kg m^−2^ s^−1^)
${A}^{*}$: Space-dependent coefficient
$$K$$: Darcy parameter
$Ea$: Activation energy coefficient (kg m^−2^ s^−2^)
$Sb$: Bio-convection Schmidt number
Nn: Local density of motile microorganisms
$$Rd$$: Radiation parameter
$Ma$: Marangoni convection parameter
${k}^{+}$: Mean absorption coefficient (cm^−1^)
Nu: Nusselt number
Sh: Sherwood number
$$Mn$$: Marangoni number
${q}_{r}$: Radiative heat flux (kW m^−2^)
$Rc$: Chemical reaction parameter
$$M$$: Magnetic parameter

## Greek symbols

${\rho }_{f}$: Fluid density (kg m^−3^ s)
${\gamma }_{2}$: Solutal relaxation parameter
$${\nu }_{f}$$: Kinematic viscosity (m^2^ s^−1^)
$${\sigma }_{1}$$: Surface tension (N m^−1^)
$$\tau$$: Thermophoretic parameter
${\lambda }_{T}$: Relaxation time of heat flux (W m^−2^)
${\gamma }_{\text{C}}$: Surface tension coefficients for concentration
$\delta$: Temperature difference coefficient
${\Phi }_{1}$: Volume fraction of MoS_2_
$${\sigma }_{0}$$: Constant
${\varepsilon }_{1}$: Viscosity variation exponent parameter
k_*f*_: Thermal conductivity (W m^−1^ K^−1^)
${\varepsilon }_{3}$: Variable mass diffusivity parameter
$\lambda c$: Relaxation time of mass flux (kg m^−2^ s^−1^)
${\gamma }_{T}$: Surface tension coefficients for temperature (N m^−1^)
$\sigma$: Microorganisms concentration difference parameter
${\sigma }^{*}$: Stefan-Boltzmann constant (W m^−2^ K^−4^)
${\Phi }_{2}$: Volume fraction of Ag
$${\sigma }_{f}$$: Electrical conductivity (s m^−1^)
${\mu }_{f}$: Dynamic viscosity (kg m^−1^ s^−1^)
${\gamma }_{1}$: Thermal relaxation parameter
${\varepsilon }_{2}$: Variable thermal conductivity parameter

## Superscript

′: Derivative with respect to $\xi$
$$f$$: Base fluid
$$hnf$$: Hybrid nanofluid
$$0$$: Surface
$$\infty$$: Ambient
